# Unveiling the early defense response dynamics in grapevines against *Plasmopara viticola* by single-cell transcriptomics

**DOI:** 10.1186/s13059-025-03904-z

**Published:** 2026-01-27

**Authors:** Xiukun Yao, Zhizhuo Xu, Yasheng Xi, Xinyue He, Qifei Gao, Jiang Lu, Peining Fu

**Affiliations:** 1 Center for Viticulture and Enology, School of Agriculture and Biology, Shanghai Jiao Tong University, Shanghai, China; 2 School of Agriculture, Food and Ecosystem Sciences, Faculty of Science, University of Melbourne, VIC, Australia

**Keywords:** Grapevine, Grape downy mildew, Plant-pathogen interaction, Single-cell transcriptome, Spatial transcriptome, Guard cell, Stomatal immunity

## Abstract

**Background:**

The viticulture has long suffered from the downy mildew caused by *Plasmopara viticola*, a strictly obligate biotrophic oomycete. Numerous studies have been performed to reveal how grapevine defends against *Plasmopara viticola*, but they mainly investigate the plant defense responses on the whole tissue level, not on the cellular level.

**Results:**

Here we employ single-cell RNA sequencing and spatial RNA sequencing to profile approximately 100,000 individual cells (~ 89,000 from scRNA-seq and ~ 11,000 from spRNA-seq), generating the first single-cell transcriptome atlas of grapevine leaves during *Plasmopara viticola* infection. This high-resolution atlas reveals the dynamic and distinct defense responses of plant cells at early stages of oomycete infection. Notably, we find that *Plasmopara viticola* reprograms the guard cell transcriptome to facilitate successful invasion, likely by altering the expression of ABA negative regulators and modulating a potassium channel regulatory pathway to influence stomatal movement.

**Conclusions:**

Overall, our work reveals differential and dynamic responses of grapevine to the *Plasmopara viticola* infection at a single-cell level, providing valuable clues for dissecting the interaction between plants and oomycetes.

**Supplementary Information:**

The online version contains supplementary material available at 10.1186/s13059-025-03904-z.

## Background

The cultivated grapevine (*Vitis vinifera* L.) is a world-wide economically important perennial fruit crop, and downy mildew caused by *Plasmopara viticola* is one of the most devastating diseases for grapevine. Current feasible managements of downy mildew rely on the massive use of chemical compounds, resulting in high economic costs and negative impacts on human health and the environment. Thus, there is a great urge for researchers to uncover the *P. viticola* infection mechanism and thereby to find sustainable strategies for disease control.

The life cycle of *P. viticola* has been well described previously [1–3]. Briefly, it consists of two stages: a sexual cycle and an asexual cycle. Oospores produced during the sexual cycle enable *P. viticola* to overwinter in leaf litter and soil, ensuring its survival until the next growing season. While the asexual cycle is the primary phase responsible for severe reductions in grape yield and quality, as zoospores generated during this stage drive rapid infection. Upon encountering the water film on the abaxial surface of grape leaves, zoospores actively swim toward stomata and begin developing a germ tube, which penetrates the stomata at approximately 6 h post-inoculation (hpi). The first haustoria, essential for further colonization, form around 24 hpi. As the hyphae continues to expand, sporangia develop between 5 and 7 days post-inoculation (dpi), releasing zoospores that initiate a new infection cycle. The dynamic interaction between grapevine and *P. viticola* could also be explained with the “zigzag” model [4, 5]. Pathogen-associated molecular patterns (PAMPs) from *P. viticola* are perceived by grapevine pattern recognition receptors (PRRs) situated in plasma membranes, triggering the PAMP-triggered immunity (PTI). As a countermeasure, *P. viticola* suppresses the PTI signaling pathway by secreting effector proteins, including RxLR proteins, CRN (crinkling and necrosis-inducing or Crinkler) proteins and YxSL[RK] proteins [2, 6–10]. In response, grapevine develops an effector-triggered immunity (ETI), which involves the perception of specific effectors either directly or indirectly by nucleotide-binding site and leucine-rich repeat (NLRs).

To understand how *P. viticola* reshapes the grapevine transcriptome, many bulk RNA-seq assays have been performed [11–16]. However, these bulk RNA-seq approaches only obtain the average gene expression across entire tissues, potentially missing important cell-type-specific transcriptomic reprogramming. Taking the grapevine-*P. viticola* interaction as an example, the invasion of *P. viticola* is through grapevine stomata, and thus the immune response of the guard cells has received considerable attention. It has been reported that stomata exhibit bigger apertures in *P. viticola*-colonized areas compared to healthy leaves, facilitating pathogen infection. Interestingly, this stomatal lock-open phenomenon is not attributed to mechanical forces exerted by the pathogen in the substomatal cavity, suggesting that the pathogen may actively inhibit stomatal closure or induces reopening by modulating the transcriptional reprogramming of guard cells [17]. However, guard cells only compose a tiny part of the whole leaf, and the guard cell transcriptomic changes will be masked by other cells in the bulk RNA-seq.

Single-cell RNA sequencing (scRNA-seq) has emerged as a powerful tool to overcome these limitations, because it can detect specific cell states and differential responses among various cell types, dissecting transcriptomic reprogramming in the process of cell development, differentiation and different cell fate determination [18–20]. For plant-pathogen interactions, scRNA-seq facilitates the identification of distinct transcriptional patterns associated with different stages of infection, revealing cellular heterogeneity within tissues that plays critical roles in pathogen defense and susceptibility [21–25].

In this study, we used scRNA-seq to investigate grapevine single-cell transcriptomic responses to *P. viticola* infection and established the first single-cell leaf transcriptome atlas of grapevine during *Plasmopara viticola* infection. The atlas enabled the identification of pathogen-responsive cell clusters at strong immune state, basal immune state, and susceptible state. Moreover, we found a down-regulation of ABA signaling and a potassium channel regulation pathway (WRKY41-WRKY55-KAT1) may be involved in stomata opening induced by *P. viticola*.

## Results

### Single-cell leaf transcriptome atlas of grapevine during *Plasmopara viticola* infection

*Vitis vinifera* cv. Cabernet Sauvignon, a widely cultivated variety for wine-making, is very susceptible to *P. viticola*, providing a compatible plant–microbe interaction model for our study. To gain insights into how *P. viticola* infects the grapevine, we employed the scRNA-seq to profile the transcriptomic changes of massive individual grapevine leaf cells.

**Fig. 1:**
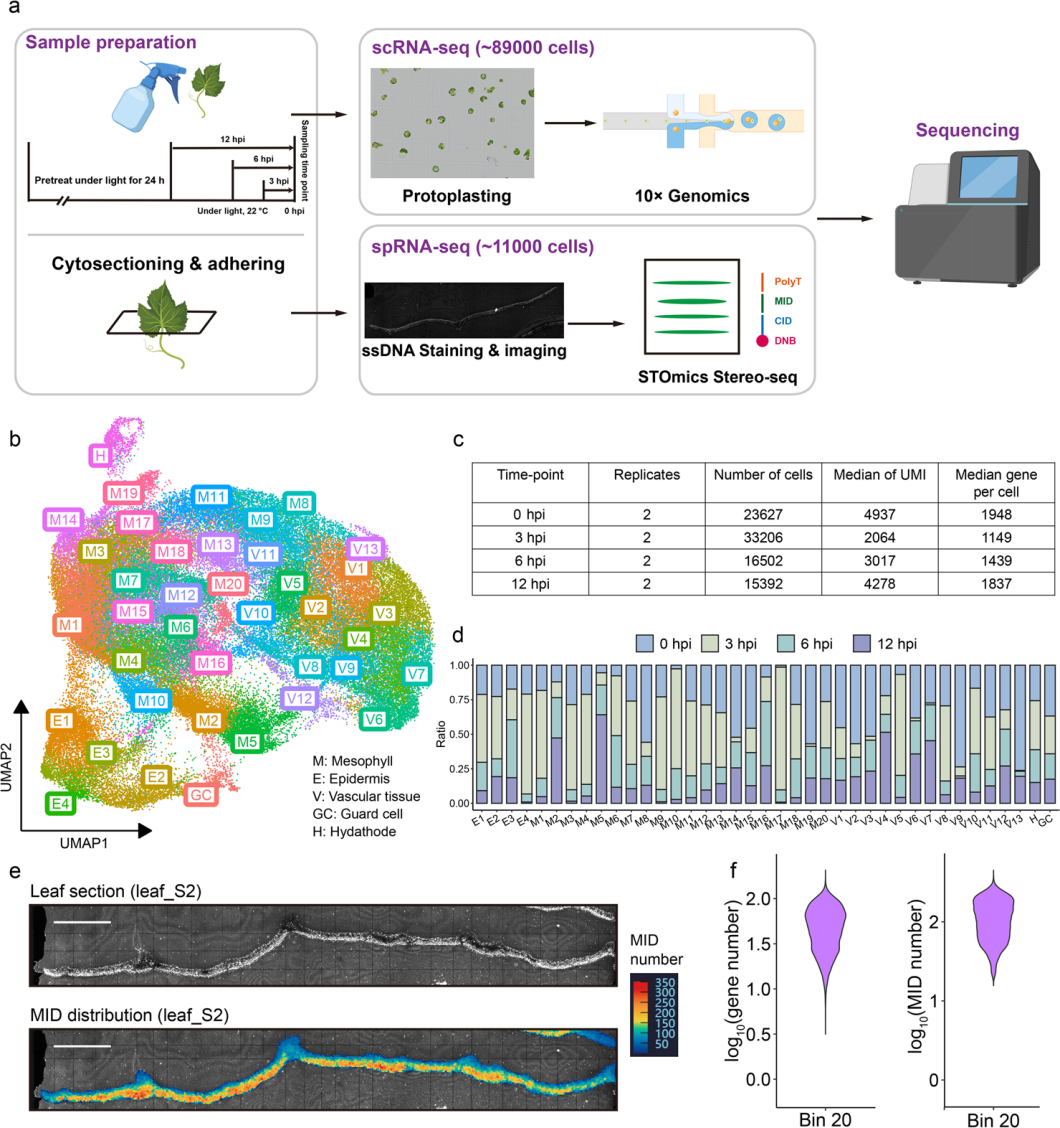
Single-cell RNA-seq profiling of *Vitis vinifera* cv. Cabernet Sauvignon infected with *Plasmopara viticola*. **a** Overview of the experiment design and procedure. For scRNA-seq, eight-week-old *Vitis vinifera* cv. Cabernet Sauvignon was pretreated under continuous light for 24 h to control transcriptome reprogramming caused by circadian rhythm, then *Plasmopara viticola* spore suspension was sprayed at different time points, all protoplasts were prepared at the same time. Each treatment sets two biological replicants. For spRNA-seq, *V. vinifera* cauline leaves were embedded in OCT and cryo-sectioned. **b** Single-cell transcriptome atlas of grape leaves infected by *P. viticola*, visualized by uniform manifold approximation and projection (UMAP) plot, colored according to cluster identities. **c** A summary of processed scRNA-seq data at each time point. Unique molecular identifier (UMI), transcripts detected per cell. **d** Proportions of cells from each time point samples in each cluster. **e** *Vitis vinifera* cauline leaf (leaf_S2: leaf section #2) showing the spatial distribution of MIDs. The color bar indicates the number of MIDs, and the scale bar represents 500 μm. **f** Violin plots showing the distribution of gene counts and MID counts per Bin 20 (20 × 20 DNB bins) across all leaf samples

Based on previous studies [1, 11], zoospores of *Plasmopara viticola* were seen at stomata and expressions of several RxLR effectors were recorded by 12 h post-inoculation (hpi), indicating that the plant’s initial immune response might have already been compromised at this stage. To capture the early events of pathogen infection prior to this immune suppression, we selected four time points, 0, 3, 6, and 12 hpi, as profiling stages of early infection. Two biological replicates were included for each time point to ensure data reliability. Protoplasts isolated from leaf tissues of eight samples were subsequently captured and analyzed with the 10 × Genomics Chromium platform (Fig. 1a). After applying quality control measures, minimizing batch effects and aligning the sequencing reads to the published genome of *V. vinifera* cv. Cabernet Sauvignon [26], a total of 88,727 cells were found in our scRNA-seq dataset, and 22,022 protein-coding genes were detected, representing 79.53% of annotated genes in grape cultivar Cabernet Sauvignon. On average, 1,593 genes and 3,574 unique molecular identifiers (UMIs) were detected in each cell (Fig. 1c; Addition file 1: Fig. S1a, b). Clustering the scRNA-seq dataset by a graph-based unsupervised analysis, we generated a transcriptome atlas, visualized on a uniform manifold approximation and projection (UMAP) plot, which contains 39 cell clusters (Fig. 1b; Addition file 1: Fig. S1c, d).

### Predicting cluster annotations by integrating cross-species OMGs and spRNA-seq dataset

Since there are no reports on cell heterogeneity in grapevine, to annotate these clusters, cross-species Orthologous Marker Gene Groups (OMGs) method and spRNA-seq location-based annotation were integrated to identify cell-type specific marker genes [27–30]. For OMGs method, scRNA-seq datasets from four species, the well-annotated model plant *Arabidopsis thaliana* [23, 24], a wood plant rubber tree *Hevea brasiliensis* [22], horticultural crops woodland strawberry *Fragaria vesca* [21] and tomato *Solanum lycopersicum* [25], were used for reference datasets. High confidence marker genes from these datasets were manual curated by and extracted from PlantscRNAdb [31], orthologous marker genes in *V. vinifera* were identified as candidate marker genes. For the spRNA-seq dataset, sequencing reads were aligned to the *V. vinifera* reference genome, and spatial transcriptomic data were integrated with stained images. Transcriptome information was extracted from individually sampled cells based on the coordinates of clustered bins (Bin 20, 20 × 20 DNB bins). Leaf sections and MID (molecular identifier) distributions were visualized, and low-MID coverage regions were excluded from downstream analysis (Fig. 1e; Additional file 1: Fig. S2b). The number of detected genes and MIDs per section and across all tissues was comparable to those reported in *Arabidopsis* leaf spatial transcriptomics [27], the number between genes and MIDs was also highly correlated, indicating that the sequencing quality was sufficient for further analysis (Fig. 1f; Additional file 1: Fig. S2c-e). In total, 11,054 cells were identified in the spRNA-seq dataset. Based on spatial localization, four cell types were annotated, and corresponding cell-type-specific marker genes were identified (Additional file 1: Fig. S4b). Leveraging marker genes identified by the two methods, we assigned a specific cell-type identity to each cluster, five types of tissue were annotated: mesophyll, epidermis, vascular tissue, guard cell and hydathode (Fig. 1b; Additional file 1: Fig. S4a; Additional file 2: Table S1). We further validated the reliability of the five identified cell types from two aspects. First, we examined the expression of previously reported marker genes within each cell type. For example, *OPEN STOMATA 1* (*OST1*) and *MITOGEN-ACTIVATED PROTEIN KINASES 12* (*MPK12*) are specifically expressed in guard cell, functioning in abscisic acid (ABA)-mediated stomatal response [32, 33]. *SMALLER TRICHOMES WITH VARIABLE BRANCHES* (*SVB*) is regulating trichome formation on epidermal surface [34]. Second, the cell type annotations were further validated through GO enrichment analysis of tissue-specific genes. The enriched biological processes in each cell type were consistent with their known physiological functions, supporting the reliability of the annotations (Additional file 1: Fig. S4d). For example, guard cells were enriched in guard mother cell differentiation, stomatal lineage progression and guard cell differentiation. Epidermis were enriched in cuticle development, secondary metabolite biosynthetic process, while mesophyll were enriched in photosynthesis and light reaction as reported [27]. Vascular cells were enriched in phloem development and xylem development. Hydathode were enriched in defense response GO terms such as response to chitin, response to wounding. The hydathode has been reported as an immune gate that protects leaf vasculature from pathogen invasion [35]. Given that our scRNA-seq dataset captures plant–pathogen interactions, the observation of this result is both reasonable and expected. A cross-library assessment of cell-type annotation replicability was also performed, revealing highly correlated AUROC scores for the annotated cell types across the experimental libraries (Addition file 1: Fig. S5). These results demonstrated the reliability of cell-type identification using cross-species OMGs and spRNA-seq dataset.

The cell numbers of each cluster varied from 164 (V13) to 8,605 (M1) (Fig. 1b). 20 clusters (briefly M1 to M20) were assigned as mesophyll cells, representing approximately 61% of the total cells. They are followed by vascular tissue (13 clusters, V1 to V13, 28% of the total cells), epidermis (4 clusters, E1 and E4, 10% of the total cells), hydathode (1 clusters, H, 1% of the total cells) and guard cells (1 clusters, GC, 0.6% of the total cells). We further examined the distribution of cells from different treatments within each cluster and observed significant variation across clusters. For instance, clusters M19, V9, and V13 were predominantly composed of cells from the 0-hpi sample, while clusters M17, V5, and E4 primarily contained cells from the 3-hpi sample. Clusters M2, M5 and V4, on the other hand, were mainly populated by cells from the 12-hpi sample (Fig. 1d).

These results indicate that the specific transcriptome signatures and the clustering of cell populations were primarily determined by cell type and responses to different oomycete infection stages. In summary, we constructed a single-cell transcriptome atlas encompassing all major cell types in grapevine leaf tissues, capturing dynamic gene expression changes at early stage of downy mildew disease development.

### scRNA-seq reveals distinct plant defense responses at early infection stage

**Fig. 2:**
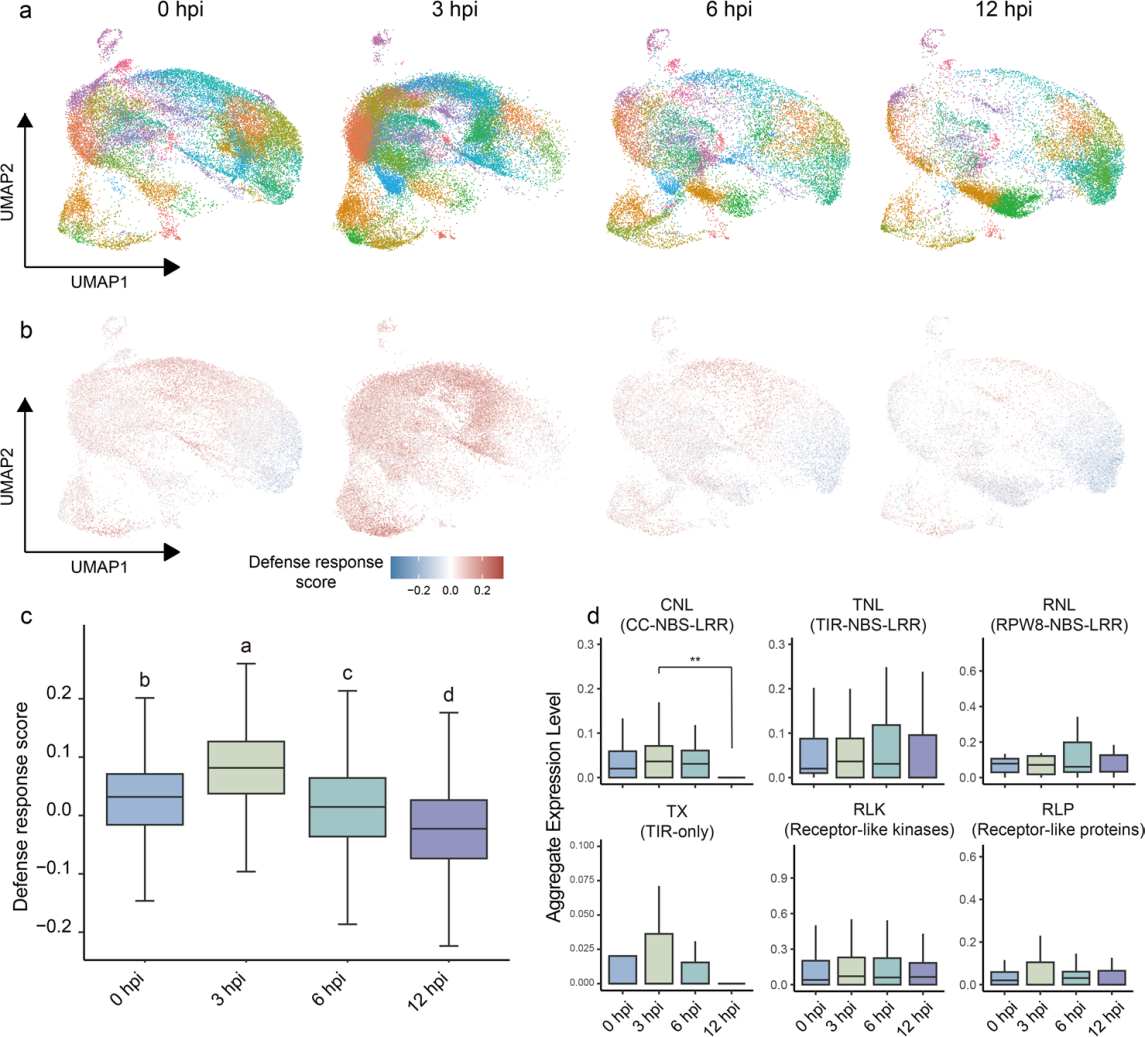
Defense score shifting along infection process. **a** Separate single-cell atlas generated from tissues at different time points. **b** Defense scores of each time point corresponding to panel **a**, **c** Quantification and multiple comparison of defense scores of four time-points. The tops and bottoms of boxes represent the 75th and 25th percentiles, respectively. Different letters above bars indicate significant differences determined using Kruskal–Wallis test with Wilcoxon rank-sum test (*p* < 0.05). **d** Expression level of RGAs in guard cells. *P*-value was calculated by Kruskal–Wallis test with Wilcoxon rank-sum test. Double asterisk (**) means *p* < 0.01

A cell cluster proportion shift could be clearly observed in the separate single-cell atlas generated from tissues of different time points (Fig. 2a). To explore whether defense response drove this shift or not, a defense response score was calculated based on gene expression modules for gene sets known to be involved in regulation plant immunity positively or negatively (Additional file 2: Table S2). We found cell clusters enriched in the 3-hpi single-cell atlas exhibited higher defense response score, while clusters enriched in the 12-hpi atlas showed lower defense response score, and the 0-hpi and 6-hpi clusters displayed similar basal levels of defense response (Fig. 2b, c), supporting the notion that defense response reshapes the cell clusters proportion.

To avoid influence of different cell types, we also separately calculated the defense response scores in different cell types and found similar trends (Additional file 1: Fig. S6a). These results indicated that the plant immune response was initially at basal level (0 hpi), and strong immune response occurred at 3 hpi; then the immune response was gradually suppressed by *P. viticola* and shifted toward susceptibility over time (6 hpi), and ultimately reached a fully susceptible state at the later infection stage (12 hpi). These single cell transcriptomic changes were consistent to the compatible plant–microbe interaction progress, in which plant defenses undergo an immune-to-susceptible state transition [24].

Given the essential role of immune receptors in plant defense [4, 5, 36], we then analyzed expression patterns of NLRs and PRRs across various cell types and different infection stages. To identify the resistance gene analogs (RGAs) in the genome of grape cultivar Cabernet Sauvignon, we employed the RGAugury [37] and identified 791 NLRs, including 98 TXs (TIR-only), 143 TNLs (TIR-NBS-LRR), 518 CNLs (CC-NBS-LRR), and 32 RNLs (RPW8-NBS-LRR), along with 2,270 PRRs, consisting of 377 RLPs (receptor-like proteins) and 1,893 RLKs (receptor-like protein kinases). Among these RGAs, 552 could be detected in our scRNA-seq atlas, comprising 27 TXs, 65 CNLs, 24 TNL, 7 RNLs, 53 RLPs and 376 RLKs (Additional file 2: Table S3).

We found that in all five tissues (mesophyll, epidermis, vascular tissue cells, guard cell and hydathode), the CNLs were all significantly down-regulated at 12 hpi compared to 3 hpi. The expression of CNLs and TXs in guard cell were dramatically reduced to near 0 at 12 hpi (Fig. 2d), indicating *P. viticola* fully overcome the stomatal immunity at the end of early infection stage. And in some tissues like mesophyll and vascular tissue, the CNLs were up-regulated significantly at 3 hpi and gradually reduced along time. For tissues like epidermis and hydathode, the expression of CNLs at 12 hpi were even lower than 0 hpi, suggesting that CNLs mediated ETI were highly suppressed in these cells (Additional file 1: Fig. S6b).

As RLKs and RLPs are receptors recognizing various pathogen-associated molecular patterns (PAMPs), their expression is vital for activating plant immunity. In mesophyll and vascular tissue, RLKs and RLPs were gradually up-regulated from 0 to 6 hpi, and then down-regulated, while in hydathode, the expression peak of RLKs and RLPs appeared at 3 hpi. In epidermis and guard cells, there were no significant changes among different time points (Additional file 1: Fig. S6c).

### Temporal transcriptional reprogramming of guard cells during *P. viticola* Infection

**Fig. 3:**
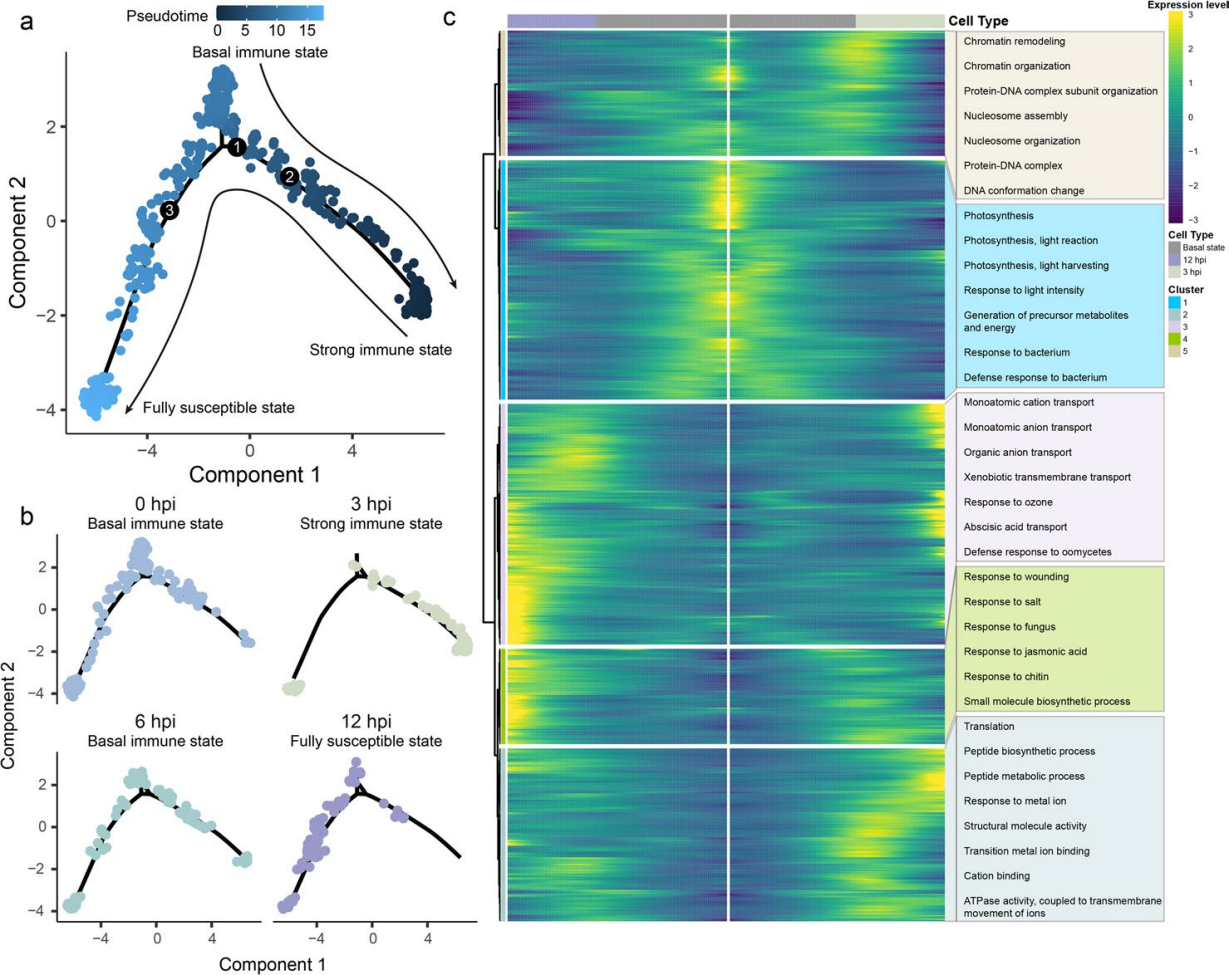
Spatiotemporal heterogeneity of guard-cell-specific cellular response to pathogen invasion. **a** Pseudo-time trajectory of guard cells. Each dot represents a single cell. Color represents the pseudo-time score. Arrows indicated the hypothesized trajectory shift based on actual pathological process. **b** Guard cell distribution on the trajectory for 0 hpi, 3 hpi, 6 hpi, 12 hpi samples. **c** Different cellular processes are enriched in cell populations that have different pseudo-time values. The heatmap shows relative expression levels of pseudo-time-dependent genes in the trajectory of guard cells, which were grouped into five populations based on hierarchical clustering. Representative gene ontology (GO) terms enriched in each population are highlighted

Since *P. viticola* invades the grapevine through stomatal pores [3, 7], we paid special attention to the transcriptomic changes of guard cells. Because the guard cell clusters in our scRNA-seq atlas contained cells from variable defense response stages, our experiments should have captured a representative snapshot of their defense activity. We utilized Monocle2 [38] to perform a pseudo-time analysis, revealing a linear order of cells. Then, the linear order of cell pseudo-time trajectory was visualized (Fig. 3). The linear cell trajectory revealed three branches, indicating two different cell fates originating from the root branch (branch 1) (Fig. 3a). Additionally, the trajectory analysis across separate time points showed that guard cells at 3 hpi and 12 hpi were distributed along two different branches, suggesting distinct defense responses. Guard cells at 0 hpi and 6 hpi displayed a similar distribution, which meant a similar basal defense response pattern (Fig. 3b). Combining with previous results (Fig. 2), the trajectory showed a contiguous path in which cells at 3 hpi were positioned at the beginning of the trajectory (defined as strong immune state), and 0 hpi and 6 hpi were in the middle state (basal immune state), while the immune responses in the guard cell ended at 12 hpi (fully susceptible state) (Fig. 3a, b). Considering the actual pathological process, we hypothesize that the plant defense state starts in the basal immune state. Defense-related genes are induced at the onset of the pathogen, and the trajectory shifts from the basal immune state to the strong immune state. As the pathogen suppresses plant immunity by some mechanisms, the plant immune state gradually returns to the basal immune level and is further suppressed to reach the susceptible state (Fig. 3a).

To gain more insight into the plant immunity state transition process, we simply defined this cellular trajectory as the progression from the basal immune state to two distinct immune states, then visualized significantly differential expressed genes along two branches. These genes were clustered into 5 groups based on their expression patterns (Fig. 3c; Additional file 2: Table S4). Genes predominantly expressed in the basal state of the trajectory (gene cluster 1) were mainly enriched in biological processes related to photosynthesis and light response. For the 3 hpi branch (strong immune state), gene cluster 5 gradually became activated, with enrichment in chromatin remodeling and organization, suggesting a potential role of epigenetic modifications in the plant defense response to pathogens. Subsequently, genes in cluster 2 were highly expressed at 3 hpi, primarily involved in cation and metal ion-related processes, as well as peptide biosynthesis. Correspondingly, receptor genes also had higher expression level at 3 hpi than other time points (Fig. 2d), suggesting activation of pattern-triggered immunity (PTI) or effector-triggered immunity (ETI). For the 12 hpi branch (fully susceptible state), cluster 3 and 4 were enriched genes associated with responses to various biotic and abiotic stresses, as well as hormones like abscisic acid and jasmonic acid which are key regulators of stomatal movement.

Our analysis reveals dynamic stepwise temporal progression in guard cells defense response. The pseudo-time trajectory precisely reflects the temporal expression changes of individual genes during plant immunity process, providing a refined view of the changes that a cell undergoes during its transition from immunity state to susceptible state.

### Identification of cluster-specific marker genes and associated modules involved in guard cell defense response to *P. viticola*

**Fig. 4:**
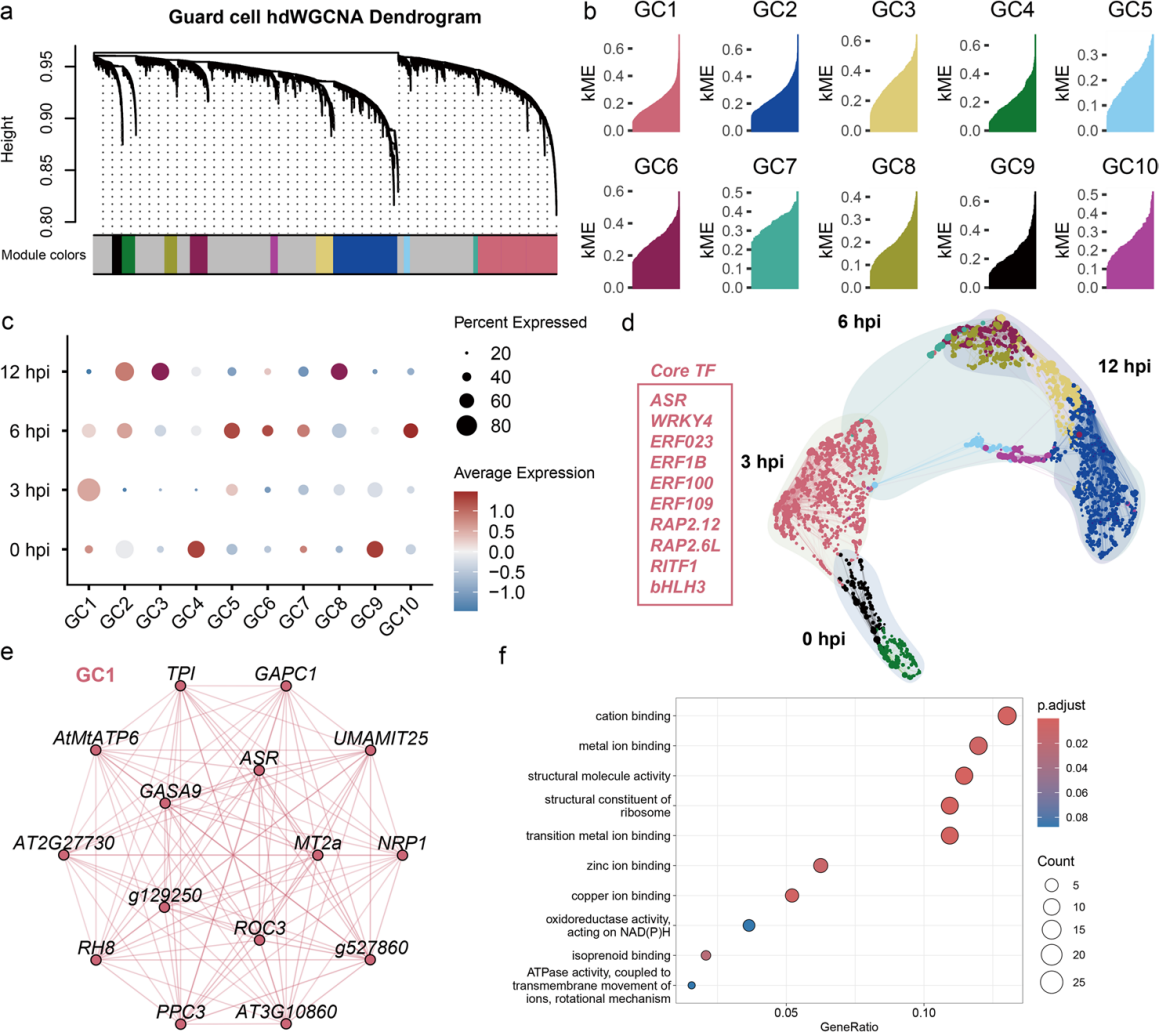
hdWGCNA analysis of guard-cell-specific genes involved in response to pathogen. **a** Guard cell hdWGCNA Dendrogram. The dendrogram shows the different co-expression modules resulting from the network analysis. Each leaf on the dendrogram represents a single gene, and the color at the bottom indicates the co-expression module assignment. **b** kME for each gene in each module. The genes in each module were ranked by kME. **c** The dot plot of expression level of modules in each time points samples. The size of the circle means the percentage of expressed genes. **d** Low-dimensional UMAP of co-expression networks of different time point samples. 10% of the edges are kept in this network. **e** GC1 module network. The top 10 hub genes by kME are placed in the center of the plot, while the remaining 15 genes are placed in the outer circle. **f** The GO enrichment (biological process) of GC1 module hub genes. The circle size represents enriched gene numbers. Red and blue represent significant and insignificant enrichments, respectively

We applied high-dimensional weighted gene co-expression network analysis (hdWGCNA) to group cluster-specific marker genes of guard cells into modules [39]. A total of 10 modules were identified (named GC1 to GC10), and each module was assigned a unique color identifier, and the poorly connected genes colored in gray will be ignored for further analysis (Fig. 4a). To identify hub genes in each module, the kME (eigengene-based connectivity) of all genes was calculated, and genes were visualized in rank order by kME within each module (Fig. 4b).

To determine the time points of gene expression represented by each module, we examined the percentage of expressed genes and average expression level within every module. The results showed that GC4 and GC9 were associated with 0 hpi, GC1 was predominantly expressed at 3 hpi, GC5, GC6, GC7, and GC10 at 6 hpi, while GC2, GC3, and GC8 were primarily expressed at 12 hpi (Fig. 4c). To explore the relationships among these modules, we utilized UMAP method to visualize all the modules together to construct a joint network. The results revealed a continuous network connectivity from 0 to 12 hpi. Although 0 hpi and 6 hpi displayed similar defense responses and were grouped into the same branch in previous pseudo-time trajectory analysis (Fig. 3b), the UMAP plot showed different co-expression network patterns between them: 0 hpi connected directly to 3 hpi, whereas 6 hpi linked both 3 hpi and 12 hpi (Fig. 4d).

Since GC1 module represented the immunity stage, we focused primarily on hub genes within the GC1 module (3 hpi). Among these genes, we observed an enrichment of oxidative stress-related genes, including transcription factor *RITF1* (RSA1 INTERACTING TRANSCRIPTION FACTOR 1), *GRXC2* (GLUTAREDOXIN C2), *TPX1* (THIOREDOXIN-DEPENDENT PEROXIDASE 1), *GAPC1* (glyceraldehyde 3-phosphate dehydrogenase) [40], *TPI* (triosephosphate isomerase) [41], *MT2a* (metallothionein-like protein 2a) [42], *USP17* (Universal stress protein 17) [43] (Fig. 4d, e). The enrichment of oxidative stress-related genes indicates a ROS burst occurring, which is a hallmark of plant immunity, at the start of early defense response. We also found that ABA-related genes were enriched in this module, which is closely linked to the regulation of stomatal movement. These include the transcription factor *ASR* (ABA stress-ripening protein), ABA receptor *PYL2*, *PYL4* and *PYL6*, and *ROC3* (ROTAMASE CYP 3), which directly regulates ABA-induced stomatal closure (Additional file 2: Table S5) [44]. Furthermore, the GO enrichment analysis revealed that cation binding and metal ion binding were the most enriched molecular functions, consistent with the findings from the pseudo-time trajectory analysis (Figs. 3c, 4f).

To explore the transcription regulator network in GC1, we listed the core transcription factors (Fig. 4d). Except ABA-related transcription factor *ASR*, a bunch of transcription factors from ERF family, including *ERF023, ERF1B, ERF100, ERF109, RAP2.6L, RAP2.12*, participated in the regulation network. Moreover, the second highest connectivity transcription factor, *WRKY4*, has been reported that positively regulated plant defense to fungal *Botrytis cinerea* but had negative impact on plant defense to bacteria *Pseudomonas syringae* [45]. Besides *RITF1*, there is another bHLH family transcription factor, *bHLH3*, which negatively regulates jasmonate signaling [46].

In representation of the 0 hpi stage, genes in modules GC4 and GC9 were primarily enriched in photosynthesis-related biological processes, with a cellular component focus on the chloroplast, consistent with findings from the pseudo-time trajectory (Fig. 3c; Additional file 1: Fig. S8). At 6 hpi, acting as a bridge between 3 and 12 hpi, four modules (GC5, GC6, GC7, and GC10) displayed distinct expression patterns and unique GO term enrichments (Additional file 1: Fig. S8). Modules GC2, GC3, and GC8 were situated at the terminal nodes of the co-expression network representing the end stage of the plant early defense response (12 hpi). Genes in GC2 were enriched in processes related to ROS response, jasmonic acid metabolism, and abscisic acid transport, consistent with the pseudo-time trajectory findings (Fig. 3c; Additional file 1: Fig. S8). In GC3, genes were enriched in nucleosome organization and protein-DNA complex assembly, suggesting a role for epigenetic modifications. For GC8, the presence of ubiquitin-related hub genes, including *UBC8*, *UBC28*, and *PUB21*, along with GO terms involving in the endosomal sorting complex required for transport (ESCRT) complex, indicated that ubiquitination and autophagy process played a role in the end stage of the plant early defense response (Additional file 1: Fig. S8).

### A guard cell-specific response reveals stomatal dynamics during pathogen early infection

Stomata serves as the battleground between grapevine and *P. viticola*. Our findings revealed that abscisic acid (ABA) transport, closely associated with stomatal closure, was enriched in both the pseudo-time trajectory and hdWGCNA analyses during the end stage of the plant-pathogen early interaction process, also lots of hub genes related with ABA enriched in module GC1. This prompted us to investigate the expression changes of ABA regulatory genes in guard cells throughout the pseudo-time trajectory.

**Fig. 5:**
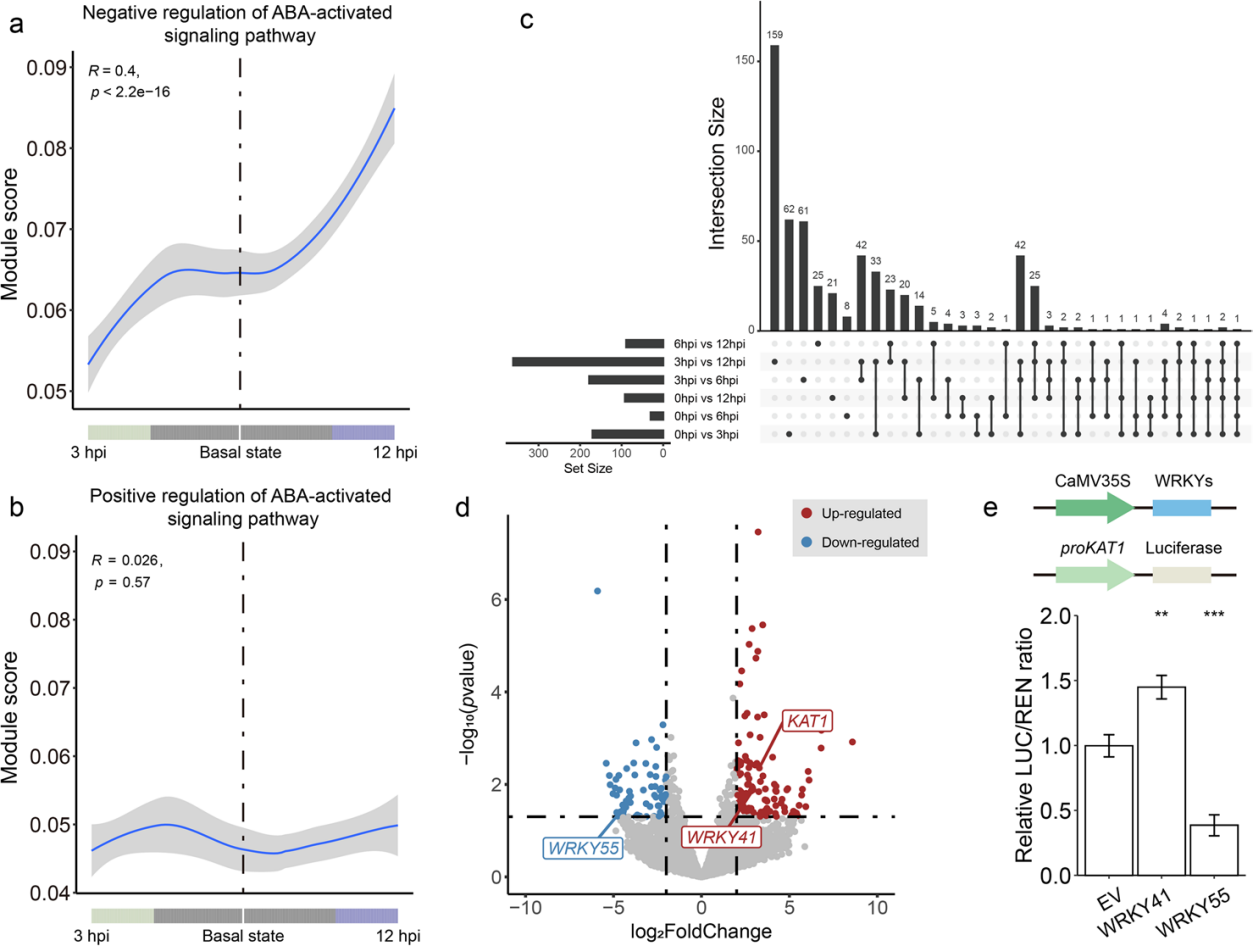
A guard-cell-specific response leads to stomatal movement under the pathogen infection. **a**, **b** ABA regulation and signaling in guard cells with two pseudo-time branches. The line plot shows linear regression of relative expression levels of genes encoding negative (**a**) and positive (**b**) regulation of ABA in guard cells, with the pseudo-time branches of these cells. **c** Venn plot of gene sets in different comparison groups, with 3 hpi vs. 12 hpi has the highest number of DEGs, while 0 hpi vs. 6 hpi with the lowest number of DEGs, consistent with previous results. **d** The volcano plot of differential expressed genes between 3 hpi and mock in guard cells. One dot indicated one gene. The red means up-regulated and blue means down-regulated. **e** The schematic plot of vectors used in the DUAL-LUC experiment. The result indicated that *WRKY41* can promote *KAT1* expression, while *WRKY55* can suppress *KAT1* expression. Error bars depict the mean ± s.e. All p values were determined by two-tailed Student’s t-test

We analyzed a total of 109 ABA regulator genes in the genome, including 62 negative regulators and 47 positive regulators (Tables S6 and S7). Interestingly, we observed that ABA negative regulator genes were down-regulated from the basal state to immune state (3 hpi), and then up-regulated from the basal state to susceptible state (12 hpi) (Fig. 5a). In contrast, ABA positive regulator genes remained stable throughout the infection (Fig. 5b). Taken together, these results suggest that ABA signaling is activated early in response to *P. viticola* infection through the down-regulation of negative regulators, then subsequently suppressed at the end stage of early defense response by the up-regulation of negative regulators in a spatially dynamic manner, which is consistent to the observation that the stomata would first close at early infection stage and then gradually re-open during the later stages.

Additionally, GO terms enriched in both the pseudo-time trajectory and hdWGCNA analyses at 3 hpi included cation binding and metal ion-related processes (Figs. 3c, 4f), which caught our attention as ion uptake and efflux is also one of the ways in which guard cells control the opening and closing of stomata. To investigate potential causal genes further, we conducted pseudo-bulk differential expression analysis across different time points. The Venn plot showed that the comparison between 3 and 12 hpi yielded the highest number of unique differentially expressed genes (DEGs), with 159, followed by 0 hpi vs. 3 hpi with 62, and 3 hpi vs. 6 hpi with 61 (Fig. 5c). We focused on DEGs from the 0 hpi vs. 3 hpi comparison. Among these genes, we found that a guard cell specific potassium channel *KAT1*, which is essential for stomatal opening [47], was significantly upregulated at 3 hpi compared to 0 hpi, with an approximately 9.8-fold change (Fig. 5d; Additional file 1: Fig. S10b), indicating *P. viticola* might induce the stomatal opening by enhancing the *KAT1* expression. However, circadian rhythms have also been reported to influence stomatal opening and closure [48, 49]. Although we pre-treated the plants under continuous light for 24 h, it was necessary to examine the expression levels of circadian rhythm-related genes. The module score for circadian rhythm remained relatively stable compared to ABA negative regulator genes and *KAT1* (Additional file 1: Fig. S9a). Additionally, the expression levels of two reported circadian clock genes, *CCA1* and *TOC1*, exhibited a steady trend throughout the infection stages (Additional file 1: Fig. S9b, c) [50]. Based on these results, it could be concluded that the transcriptional changes observed in the ABA negative regulation pathway and *KAT1*, and subsequently stomatal movement, were primarily driven by *P. viticola* infection.

To elucidate the regulatory pathway involved in the *KAT1* transcriptional activation, we cloned *KAT1* promoter and analyzed its *cis*-acting elements. One W box, a *cis-*element for *WRKY* transcription factors, was found to present in the *KAT1* promoter (Additional file 1: Fig. S10a). We then examined *WRKY* genes in the DEGs between the 0 hpi and 3 hpi comparison, and found two *WRKY* transcription factors: *WRKY41*, which was upregulated approximately 7.6-fold, and *WRKY55*, downregulated by about 24.4-fold (Fig. 5d; Additional file 1: Fig. S10c, d). In addition, we found the expression level of *KAT1* had similar trends to *WRKY41*, while it was opposite to *WRKY55* (Additional file 1: Fig. S10b-d). Using a dual-luciferase assay, we assessed their regulatory effects on *KAT1* expression. The results showed that both *WRKY41* and *WRKY55* bind to the *KAT1* promoter. Notably, *WRKY41* significantly activated *KAT1* expression, while *WRKY55* suppressed it (Fig. 5e). These findings suggest that *P. viticola* promotes *KAT1* expression by up-regulated *WRKY41* and down-regulated *WRKY55*, driving the transition of stomatal states from closure to opening, and facilitating the entry of the pathogens.

## Discussion

**Fig. 6:**
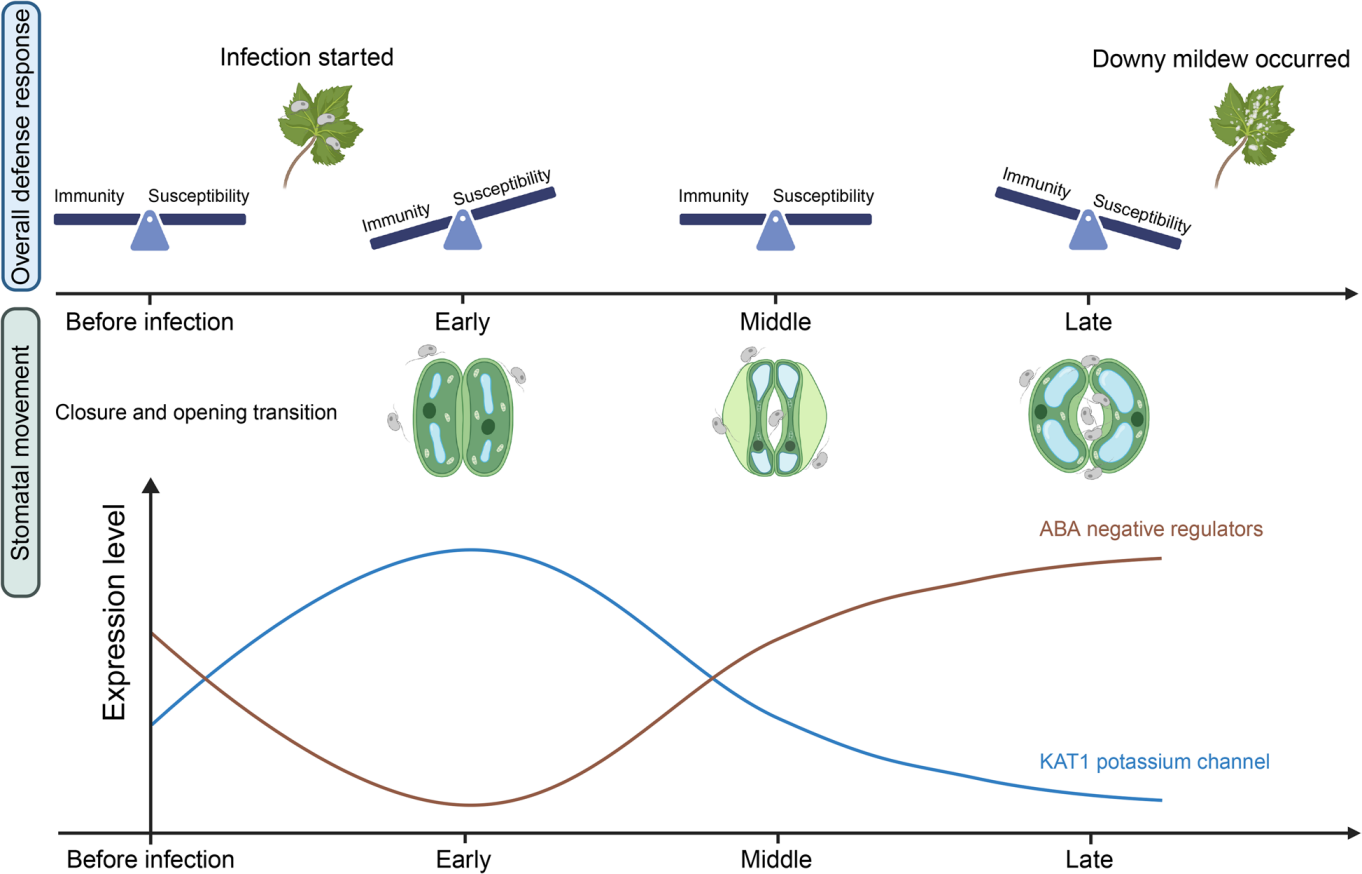
Model of defense response state transitions and stomatal movement in grapevine during *P. viticola* infection. In response to *P. viticola* infection, grapevine leaves initiate the expression of immunity-related genes, such as PRRs and NLRs, to activate immune defenses, displaying tissue-specific heterogeneity. As the infection progresses, the plant's defense response gradually shifts from immunity to susceptibility. Guard cells, as the initial points of exposure to *P. viticola* zoospores, reflect the dynamic counter-regulation between plant and pathogen through stomatal movements. Early in infection, stomata close to block pathogen entry. However, after encountering stomatal immunity, the stomata reopen, allowing pathogen invasion. This process may involve ABA negative regulators and the KAT1 potassium channel. Created with BioRender.com

Compared with the bulk RNA-seq technique which reveals transcriptional changes at the whole-tissue level, single-cell RNA-seq analyzes the transcriptomes of individual cells, and avoids the loss of information that occurs due to signal averaging in bulk RNA sequencing, thereby enhancing our understanding of when and how plants respond to various biotic stresses [21–24]. In this study, we constructed the first single cell transcriptome atlas of grape leaves responding to the infection of *Plasmopara viticola* with ~ 89,000 cells, revealed the distinct defense responses existed at early infection stage, and characterized potential signaling pathways controlling stomatal movement during this infection via multiple analysis methods (Fig. 6). Our work thus not only provides a comprehensive framework about the grapevine-*P. viticola* interaction, but also offers valuable resources for other researchers in this area.

To date, several bulk RNA-seq research have been published, and most of them focus on incompatible interactions between resistance grapevine species and *P. viticola*, trying to identify functional genes, or characterize the pathways and gene networks that participate in grape resistance response at early infection stage [11–16]. Research on compatible interactions, particularly how *P. viticola* manipulates the gene regulatory network of grapevines to counteract immune responses, remains limited. Most studies have selected 6 hpi as the primary time point to investigate the grapevine early response. However, this ‘‘early response’’ may not fully capture the initial stages of host–pathogen interaction. Our findings reveal that grapevines can sense zoospores and activate initial immune responses as early as 3 hpi (Figs. 2, 3), earlier than previously established. This earlier time point better represents the true onset of plant immune activation. It has been reported that *P. viticola* develops a germ tube to penetrate stomata at 6 hpi [3]. In our dataset, the immune system begins to be compromised by 6 hpi, as *P. viticola* intensifies its efforts to evade defenses, likely through the development of germ tubes to invade plant tissue further as reported. We speculate that the transcriptome reprogramming at 3 hpi could be diluted by bulk RNA-seq analysis. We also identified a set of genes that were induced at 3 hpi via different methods, providing more insights into early response during grapevine-*P.viticola* interaction process. Moreover, scRNA-seq allows us to analyze gene regulation networks at specific tissue level, which is not feasible for bulk RNA-seq. Understanding the dynamics of these early stages provides critical insights into the timing and mechanisms by which *P. viticola* establishes compatibility, shedding light on potential intervention strategies to enhance grapevine resistance.

As key components participant in plant immunity system, extracellular and intracellular receptor genes are always in the spotlight, including PRRs responsible for PTI and NLRs responsible for ETI. A previous study on *Arabidopsis* responding to a fungal *Colletotrichum higginsianum* infection revealed that NLRs could be highly induced in vasculature-related cells, especially TNLs [23]. Considering the TIR domain is not only shown in TNLs, and novel function of TIR-containing protein in plant immunity has been characterized [51, 52], we not only examined the expression level of NLRs and PRRs, but also TXs, in our oomycete-treated scRNA-seq dataset. However, we found that CNLs, instead of TNLs, shown various transcription reprogramming in all five tissues (Additional file 1: Fig. S6b). No statistically significant expression change was observed in TNLs, TXs and RNLs (Additional file 1: Fig. S6b, c), revealing different strategies that plant dealing with different types of pathogens. From another perspective, numerous *Resistance to Plasmopara viticola* (RPV) QTLs have been mapped onto the grape genome but only two of them, *RPV1* and *RPV3*, have been isolated and functionally validated. Both these two R genes belong to the TNL family [53, 54], we may also consider the non-induced TNLs, TXs and RNLs as a phenotype of immune suppression. For PRRs, those in the mesophyll, vascular tissue, and hydathodes were significantly induced at 3 hpi but suppressed to varying degrees thereafter. However, no significant changes in PRR expression were observed in the epidermis and guard cells (Additional file 1: Fig. S6c). Since epidermis is the first site to be exposed to *P. viticola*, and guard cells serve as the battleground between plant and pathogen, PTI may be rapidly suppressed in these tissues. Interestingly, the activation of PRRs in other adjacent tissues, combined with the enrichment of two GO terms, ‘‘peptide biosynthetic process’’ (GO:0043043) and ‘‘peptide metabolic process’’ (GO:0006518) in the guard cell trajectory branch toward to 3 hpi (Fig. 3c), we hypothesize that small peptides may function as phyto-cytokines, playing a role in activating systemic acquired resistance (SAR). No research on small peptides and SAR has been reported in grapevine-*P. viticola* interaction yet, which could be a fascinating topic.

It has been well established that pathogen could hijack key components in ABA pathway to control stomata movement to create better conditions for their infection and growth [55, 56], and also previous studies demonstrated that the ABA pathway plays an essential role in the pathogen induced stomatal closure [23]. However, little is known how *P. viticola* manipulates the grapevine stomatal movement at single-cell level. Upon *P. viticola* attack, the grapevine leaf stomata will first close (at 0 hpi – 3 hpi stage) and then re-open (after 3 hpi), suggesting that *P. viticola* can manipulate the stomatal movements to facilitate its invasion. Our scRNA-seq data uncovered a guard cell type-specific response that showed spatial dynamics. In this case, *P. viticola* could reopen the stomata by both interfering with ABA-related pathways and enhancing the K^+^ influx of guard cells to promote its invasion. Gene module expression score along cell pseudo-time trajectory revealed that ABA negative regulator was suppressed on the cell trajectory branch toward 3 hpi and activated on the branch toward 12 hpi (Fig. 5a). We also found that a novel potassium channel regulation pathway (WRKY41-WRKY55-KAT1) in grapevine, may drive the stomata shift from closure to opening. Yet, our explanation focuses solely on the plant intrinsic regulatory networks, and there are still many open questions for the interaction between *P. viticola* and grapevine. For example, how does *P. viticola* interfere with the ABA regulatory networks in plants? What is the upstream regulator of the WRKY41-WRKY55-KAT1 pathway? These questions are all worth exploring further.

Nevertheless, some limitations in experimental design should be acknowledged. To minimize the influence of circadian rhythms on stomatal movement, the plantlets were pre-treated under continuous light for 24 h prior to the experimental treatment to maintain stomatal opening. However, constant light exposure imposes physiological stress on plantlets [57], and both variations in light conditions and alterations in circadian rhythms can influence plant defense responses to pathogens [58–60]. Consequently, the transcriptional reprogramming induced by pathogen infection in this study may differ from that occurring under natural light conditions. Future research should include non-infected controls sampled at identical time points under normal light–dark cycles to better distinguish infection-specific transcriptional changes from those driven by circadian effects.

Overall, we present a comprehensive single-cell transcriptome atlas of *V. vinifera* during infection by the biotrophic oomycete pathogen *P. viticola*. This atlas reveals remarkable gene expression heterogeneity across the temporal dimension within grapevine leaf tissues. The dataset highlights the dynamic and complex cellular responses to oomycete infection, showcasing distinct defense mechanisms at various stages of infection. This high-resolution transcriptomic resource offers valuable insights that can drive future research into the molecular intricacies of plant immunity and pathogen invasion. Furthermore, the findings contribute to a deeper understanding of the biological processes underlying plant-pathogen interactions and may enhance grapevine resistance breeding efforts as well as the development of innovative strategies for managing the downy mildew.

## Conclusions

In summary, we constructed the first single-cell transcriptome atlas of grape leaves during *P. viticola* infection, profiling approximately 89,000 individual cells. By calculating defense response scores, we identified distinct immune responses from the start to the end of early infection stage, with similar trends observed across different tissues. Focusing on guard cell-specific transcriptomes, we employed pseudo-time trajectory analysis and co-expression gene network construction, uncovering how *P. viticola* reshapes the guard cell transcriptome to facilitate invasion. This includes modulating the expression of ABA negative regulators and a WRKY-KAT1 potassium channel regulatory pathway to manipulate stomatal opening. Overall, our study unveils the grapevine dynamic, cell-specific defense responses to oomycete infection, providing insights into pathogenesis at a high resolution.

## Methods

### Experimental materials and growth conditions

In vitro plantlets of *Vitis vinifera* cv. Cabernet Sauvignon were grown on half-strength Murashige-Skoog medium supplemented with 1.5% (w/v) sucrose, 0.1‰ (w/v) IBA and 0.8% (w/v) agar in a growth chamber under 16/8-h light/dark condition at 22 °C.

*Plasmopara viticola* strain ‘CVE’ was inoculated on fresh leaves of *V. vinifera* cv. Thompson Seedless at 22 °C with a 12-h light–dark cycle. After five days, 2–3 leaves colonized by P. viticola conidia were harvested into a 50 mL tube, filled with 25 mL double-distilled water. The tube was vortexed for 30 s, and the conidia suspension was then filtered through sterilized gauze to remove plant tissue. The conidia concentration was measured by a hemocytometer and adjusted to 1 × 10^5^ conidia per mL for further inoculation.

### Inoculation of *P. viticola* and grape leaves protoplast preparation for scRNA-seq

The 8-week-old in vitro grapevine plantlets were used for scRNA-seq. To eliminate the effects of circadian rhythms and light responses on transcriptional changes, plantlets were pre-treated under continuous light condition for 24 h before inoculation. For each plantlet, 1 mL of *P. viticola* conidial suspension was fully sprayed onto the abaxial surface of grape leaves to ensure thorough inoculation. Leaves were collected at 0, 3, 6, and 12 h post-inoculation for protoplast isolation. The inoculated plants were maintained under continuous light in a growth chamber at 22 °C until sampling.

The infected leaves were cut into ~ 0.5 mm thin strips and immediately transferred into protoplast enzyme solution (1.5% cellulase R10, 1.5% macerozyme R10, 1% Driselase, 0.8% Snailase, 12% (w/v) mannitol, 10 mM KCl, 20 mM CaCl_2_, 2 mM 2-morpholineethanesulfonic acid, and 0.1% BSA, pH = 5.7). Placed the samples and the enzymatic solution on a shaker, setting the speed to 50 rpm and the temperature to 26 °C. After digesting in the dark for 30 min, taking the samples out, gently pressed them into the enzyme solution using two dissecting needles. Then digesting in the dark for 1 h, using the dissecting needles to break the remaining leave strips apart softly. After final digesting for 30 min, the digested protoplast for each sample was filtered via 40 μm cell strainers (Absin, Shanghai, China) and washed with wash buffer (12% (w/v) mannitol, 10mM KCl, 2 mM 2-morpholineethanesulfonic acid, pH = 5.7) in a 50 mL sterile tube. Then the samples were centrifuged at 100 × *g* for 2 min at room temperature and discard the supernatant. The wash step was repeated two times for each sample. Then discard the supernatant, resuspended the protoplast using the remaining supernatant. The viability of protoplasts and presence of cell debris was determined by trypan blue staining.

### Single cell capture, RNA-library construction, sequencing and reads alignment

10 μL of protoplast suspension was mixed with 2 μL of 0.4% trypan blue solution, the protoplast concentration of each sample was counted and calculated using a hemocytometer. Only samples with a protoplast number greater than 1 × 10^5^ per mL were retained and proceeded to the next capture step. Immediately loaded samples into a Chromium Single Cell Instrument (10 × Genomics, Pleasanton, CA) to produce single-cell GEMs (Gel Bead-In Emulsions). The number of loaded cells was controlled at approximately 20,000 per sample, with the loading volume calculated based on the protoplast concentration determined in the previous step. Libraries were generated from the cDNAs with Chromium Next GEM Single Cell 3′ Reagent Kits v3.1 (10 × Genomics, Pleasanton, CA). The cDNA libraries were sequenced on the Illumina sequencing platform HiSeq 2000 by NovoGene Biotechnology Co., Ltd (Shanghai, China).

Genome of *V. vinifera* ‘Cabernet Sauvignon’ cl. FPS08 ver1.1 were downloaded from https://grapegenomics.com/pages/VvCabSauv/download.php and used as reference genome. The mkref command from CellRanger (Version 7.2.0) was used to build reference index. Raw reads were aligned to the genome using CellRanger count command with default settings.

### Spatial transcriptome tissue processing, in situ reverse transcription, library construction, sequencing and reads alignment

Young leaves were collected from 8-week-old in vitro grapevine plantlets. Then tissues were embedded in pre-cooled OCT (Leagen) and stored at −80°C until processed. The pre-frozen leaf tissues in OCT were transversely sectioned at 10 mm thickness. Tissue sections were adhered to the Stereo-seq chip (generated by BGI, China) surface and incubated at 37 °C for 3 min. Then, the sections were fixed in methanol and incubated for 40 min at −20°C before Stereo-seq library preparation. Where indicated, the same sections were stained with nucleic acid dye (Thermo Fisher, Q10212), mounted with glycerol and imaging was performed with a Motic Custom PA53 FS6 microscope prior to in situ capture at the channel of FITC. The chips were washed with 0.1 × SSC buffer (Thermo, AM9770) and permeabilized using permeabilization reagent working solution in 0.01N HCl buffer for 6–24 min at 37°C. Given different tissue properties, obtaining high-quality spatial transcriptomes of each tissue requires different incubating times by pepsin: 6 min for testis and spinal cord. The permeabilization time with the strongest staining fluorescence (24 min) was selected as the suitable permeabilization time (Additional file 1: Fig. S2a).

Tissue sections were mounted onto a Stereo-seq Chip T (1 cm × 1 cm). The chip was placed in methanol pre-chilled at − 20 °C for fixation (30 min). A fluorescent staining solution (Qubit™ ssDNA Assay Reagent, Thermo Fisher) was applied to the chip, and imaging was conducted using a fluorescence microscope in epifluorescence mode (FITC channel for ssDNA detection). Permeabilization was performed using a working solution of permeabilization reagent in 0.01 N HCl buffer, followed by incubation at 37 °C for 24 min. Reverse transcription was subsequently carried out to synthesize cDNA. After reverse transcription, tissue sections were washed twice with 0.1 × SSC buffer and digested with Tissue Removal buffer (10 mM Tris–HCl, 25 mM EDTA, 100 mM NaCl, 0.5% SDS) at 55 °C for 10 min. cDNA-containing chips were then subjected to Prepare cDNA Release Mix (cDNA Release Enzyme, cDNA Release buffer) treatment for overnight at 55 °C. cDNA were purified using the VAHTSTM DNA Clean Beads (0.8 ×).

100 ng of cDNA product was used for subsequent Multiple Displacement Amplification (MDA). The resulting amplified product was purified via 1X bead purification, and 100 ng of the purified product was utilized for library preparation. This was followed by dual-indexed PCR selection and quality control (QC) steps. The cDNA library was first converted into circular single-stranded DNA and amplified into DNA nanoballs (DNBs) via Rolling Circle Amplification (RCA). The generated DNBs were loaded onto a high-density DNA nanochip using grid-patterned microwells. Sequencing was performed on the MGI DNBSEQ-T7 sequencer employing Combinatorial Probe-Anchor Synthesis (cPAS) technology by Smartgenomics Technology Institute (Tianjin, China).

Raw reads were aligned to the genome following SAW pipeline (Version 8.1.3, available at https://github.com/STOmics/SAW) with parameters –kit-version = "Stereo-seq T FF V1.3" –sequencing-type = "PE75_50 + 100".

### Cross-species OMGs and spRNA-seq marker genes identification

For OMGs method, scRNA-seq datasets from four species, the well-annotated model plant *Arabidopsis thaliana* [23, 24], a wood plant rubber tree *Hevea brasiliensis* [22], horticultural crops woodland strawberry *Fragaria vesca* [21] and tomato *Solanum lycopersicum* [25], were used for reference datasets. High confidence marker genes from these datasets were collected from PlantscRNAdb [31], orthologous genes in *V. vinifera* were identified using OrthoFinder (Version 2.5.5) [61]. The genes belonging to the same orthogroups with high confidence marker genes were identified as OMGs [29].

The spRNA-seq downstream analysis was conducted by stereopy (Version 1.5.1) [62]. GEF file generated by SAW was loaded by st.io.read_gef_info and st.io.read_gef. To filter out low-quality data, cells with less than 10 genes were discarded. The dataset was normalized by the default tl.normalize_total method, then scale with tl.scale. Reducing dimensionality was carried out by tl.pca, then neighborhood graph was computed by tl**.**neighbors and tl**.**spatial_neighbors using 30 principal components. Finally, using Uniform Manifold Approximation and Projection to embed the graph in two dimensions by tl**.**umap. Obtained MID count matrix was converted into RDS file to load into Seurat for visulization and marker genes identification by FindAllMarkers, the following cutoffs were applied: log_2_FC = 1, p.adj = 0.

### Cell clustering and annotation for scRNA-seq

The scRNA-seq downstream analysis was conducted by Seurat (Version 5.0) [63]. Read10x was used to load raw matrix files generated by CellRanger, and the function CreateSeuratObject was used to build individual Seurat datasets. To filter out low-quality data, cells with less than 200 genes were discarded, and the genes present in less than 3 cells were not considered. These datasets were normalized by the default LogNormalize method, then scaled with ScaleData and integrated with IntegrateLayers and JoinLayers functions. Batch effects were corrected by HarmonyIntegration [64]. For further dimensional reducing and clustering, a series of resolution values (0.6, 0.8, 1, 1.5, 1.9, and 2) and found that a resolution of 2 yielded the most well-defined cell clusters (Additional file 1: Fig. S3). Fifty principal components were calculated for the integrated dataset, used to cluste r the cells with Louvain method at resolution of 2 and further dimensionally reduce the gene expression space using UMAP by RunUMAP function, using 50 principal components and default parameters. Cell-type marker genes identified by OMGs and spRNA-seq were used for cell type annotation, MetaNeighbor package (Version 1.18.0) was used to assess reproducibility of cell-type annotation at the cell-type level [65].

### Resistance gene analogs (RGAs) identification in Cabernet Sauvignon genome

Different types of RGAs were identified by RGAugury [37]. These RGAs were divided into two main groups: Nucleotide-binding site and leucine-rich repeat (NLR) genes and pattern-recognition receptors (PRRs). NLR genes could be further divided into four types: TIR-only (TX), TIR-NBS-LRR (TNL), CC-NBS-LRR (CNL) and RPW8-NBS-LRR (RNL). PRRs could be divided into two types: receptor-like protein kinases (RLKs) and receptor-like proteins (RLPs). Genes that had no expression in all cell clusters were filtered.

### Pseudobulk analysis and guard cells pseudobulk differentially expressed genes analysis

A pseudobulk expression value for each gene was calculated as the sum of all counts from all cells for that gene within integrated single-cell datasets. These values were then used to differentially express analysis and gene expression level visualization within specific tissues.

Pseudobulk counts matrix of guard cells were extracted and processed into DEseq2 (Version 1.46.0) package. Genes with low expressed counts were filtered. Candidate differentially expressed genes were selected with the criteria *p*-value < 0.05 and |log_2_FoldChange|> 2.

### Module score computation

The genes of annotated with Gene Ontology terms ‘‘immune response’’ (GO:0006955), ‘‘negative regulation of defense response’’ (GO:0031348), ‘‘negative regulation of abscisic acid-activated signaling pathway’’ (GO:0009788), ‘‘positive regulation of abscisic acid-activated signaling pathway’’ (GO:0009789) and ‘‘circadian rhythm’’ (GO:0007623) were retained (Additional file 2: Table S2, S6, S7, S9). Gene module score was computed using function AddModuleScore_UCell from UCell package (Version 2.10) [66]. The defense response score was defined as the score calculated from ‘‘immune response’’ module subtracted from the score calculated from ‘‘negative regulation of defense response’’.

### Guard cells trajectory inference and pseudotime analysis

Normalized expression values for transcripts in guard cells were extracted from the integrated dataset. Monocle 2 (Version 2.4.0) was employed for trajectory inference and pseudotime analysis. The function as.CellDataSet was used to convert Seurat object to Monocle object. Then differentialGeneTest function was used to detect differentially expressed genes between different groups with parameter fullModelFormulaStr = ‘‘ ~ group’’, differentially expressed genes with q-value < 0.01 were selected to build trajectory with function setOrderingFilter, and the ‘‘DDRTree’’ method was used to order the cells according to the expression pattern of selected genes. Then cell trajectory and genes pseudotime heatmap were visualized by function plot_cell_trajectory and plot_genes_branched_heatmap respectively.

### Guard cells cluster-associated modules identification

The co-expression networks for the cluster-specific marker genes were constructed using hdWGCNA (Version 0.4.00) package [39]. The function SetupForWGCNA was used to convert Seurat object to hdWGCNA object. Then we constructed metacells and normalized the resulting expression matrix using MetacellsByGroups and NormalizeMetacells. Function TestSoftPowers used to perform a parameter sweep for different soft power thresholds. To set Scale Free Topology Model Fit greater than or equal to 0.8, we tested soft power thresholds from 1 to 30 and finally picked 5 as the lowest soft power threshold to construct co-expression network (Additional file 1: Fig. S7). Then the co-expression network was constructed using the soft power threshold selected above by function ConstructNetwork. Harmonized module eigengenes were computed by ModuleEigengenes with default parameters. The eigengene-based connectivity (kME) of each gene were computed by ModuleConnectivity. The network plot of each module was visualized by ModuleNetworkPlot. Low-dimensional UMAP of co-expression networks were visualized by RunModuleUMAP.

### GO term enrichment

The Cabernet Sauvignon GO term annotation database was generated by eggnog-mapper [67] and in-house R script. Enrichment analysis of different module genes or pseodutime cluster genes was conducted by clusterProfiler (Version 4.14.0) [68].

### Vector construction and Dual-Luciferase reporter assay

Transient expression assays were performed in the tobacco (*Nicotiana benthamiana*) leaves using *Agrobacterium*-infiltration. The coding sequences of *WRKY41* and *WRKY55* were amplified from *V. vinifera* cv. Cabernet Sauvignon complementary DNA (cDNA) library and then subcloned into the pHB-GFP vector as effectors. The 2-kb promoter sequence of *KAT1* was amplified from *V. vinifera* cv. Cabernet Sauvignon genome DNA (gDNA) library and then subcloned into the pGreenII vector to produce the reporter. The empty pHB-GFP vector was used as the negative control. The resulting vectors were transferred separately into *A. tumefaciens* GV3101 strains. The *A. tumefaciens* containing the reporter and effector constructs were co-infiltrated into the tobacco leaves. After 48 h of infiltration, the tissues were sampled and the firefly luciferase (LUC) and Renilla luciferase (REN) activity were quantified using a Dual-Luciferase Reporter Gene Assay Kit (Yeasen, Shanghai, China) and measured by the Dual-Luciferase® Reporter Assay System (Promega, WI, USA). The relative luciferase activity was calculated as the ratio of LUC/REN. Six biological repeats were measured for each sample. Primers used for sequence cloning and vector construction are listed in Additional file 2: Table S10.

### Quantification and statistical analysis

Statistical analyses were performed in R (Version 4.2.2). Aggregated expression levels of RGAs and defense response scores of different tissues were statistically tested by Kruskal–Wallis test method and multiple comparisons were conducted by Wilcoxon Rank Sum test method. The relative LUC/REN values of different groups were statistically tested by ANOVA and multiple comparisons were conducted by Student’s t-test. Details of the statistical analysis can be found in the figure legends.

## Supplementary Information

Additional file 1: Figures S1-S10. This file contains all supplementary figures. Fig. S1 Single-cell RNA-seq profiling of *Vitis vinifera *cv. Cabernet Sauvignon. Fig. S2 Spatial RNA-seq profiling of leaf section of Cabernet Sauvignon. Fig. S3 Cell clustering tests for different resolution values. Fig. S4 Single-cell RNA-seq and spatial RNA-seq profiling with bulk tissue annotation and marker genes. Fig. S5 Cross-library assessment of cell type annotation by MetaNeighbor. Fig. S6 Defense scores and RGAs’ expression levels in different tissues. Fig. S7 Soft threshold testing for hdWGCNA. Fig. S8 GO enrichment analysis of hdWGCNA modules other than GC1. Fig. S9 Circadian rhythm pattern in guard cells during early infection stage. Fig. S10 Analysis of cis-acting elements on *KAT1* promoter and relative transcription factor expression level.

Additional file 2: Tables S1-S11. This file contains all supplementary tables. Table S1 Marker genes identified from cross-species OMGs method and spRNA-seq dataset. Table S2 Genes used to calculate defense response module score. Table S3 Detected RGAs in scRNA dataset from this research. Table S4 List of genes significantly differently expressed along the pseudotime trajectory and gene clusters they belonged. Table S5 Hub genes list of hdWGCNA modules. Table S6 List of genes with GO term Negative regulation of ABA-activated signaling pathway. Table S7 List of genes with GO term Positive regulation of ABA-activated signaling pathway. Table S8 List of genes significantly differently expressed between 0 hpi and 3 hpi in guard cells. Table S9 List of genes with GO term circadian rhythm. Table S10 Primers used in this research. Table S11 Gene name used in

## Data Availability

All the raw single-cell RNA sequencing data generated in this study have been deposited in NGDC (https://ngdc.cncb.ac.cn/) under the accession code PRJCA033302 [69]. Genome of V. vinifera ‘Cabernet Sauvignon’ cl. FPS08 ver1.1 could be accessible at https://grapegenomics.com/pages/VvCabSauv/download.php, released in previous work [26]. The names and corresponding gene IDs in Cabernet Sauvignon for all genes analyzed in this study are provided in Additional file 2: Table S11. Code and scripts used in the single-cell transcriptome analysis are available on Zenodo: https://zenodo.org/records/14327435 [70].
