# Supplementary material for: Unveiling the early defense response dynamics in grapevines against Plasmopara viticola by single-cell transcriptomics
Source: Genome Biol. 2026 Jan 27;27:43. doi: 10.1186/s13059-025-03904-z (PMC12918531; doi:10.1186/s13059-025-03904-z)
Supplement: Supplementary file 1 — Additional file 1: Figures S1-S10. This file contains all supplementary figures. Fig. S1 Single-cell RNA-seq profiling of Vitis vinifera cv. Cabernet Sauvignon. Fig. S2 Spatial RNA-seq profiling of leaf section of Cabernet Sauvignon. Fig. S3 Cell clustering tests for different resolution values. Fig. S4 Single-cell RNA-seq and spatial RNA-seq profiling with bulk tissue annotation and marker genes. Fig. S5 Cross-library assessment of cell type annotation by MetaNeighbor. Fig. S6 Defense scores and RGAs’ expression levels in different tissues. Fig. S7 Soft threshold testing for hdWGCNA. Fig. S8 GO enrichment analysis of hdWGCNA modules other than GC1. Fig. S9 Circadian rhythm pattern in guard cells during early infection stage. Fig. S10 Analysis of cis-acting elements on KAT1 promoter and relative transcription factor expression level. [file 13059_2025_3904_MOESM1_ESM.pdf]

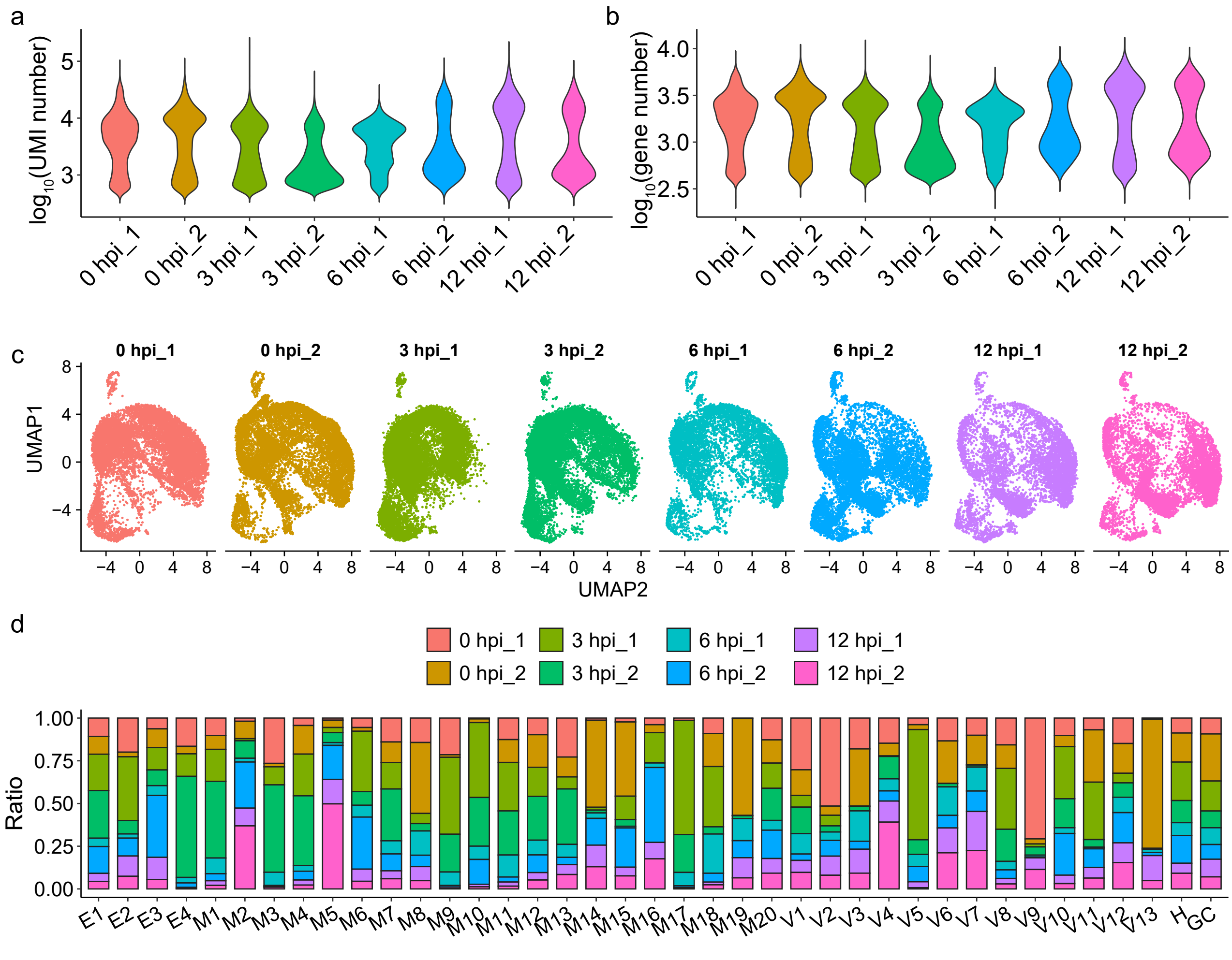

Fig. S1 Single-cell RNA-seq profiling of *Vitis vinifera* cv. Cabernet Sauvignon. a. b. Violin plots of UMI number and gene number in eight libraries. **c.** UMAP clustering for each library. **d.** Proportions of cells from each library in each annotated cluster.

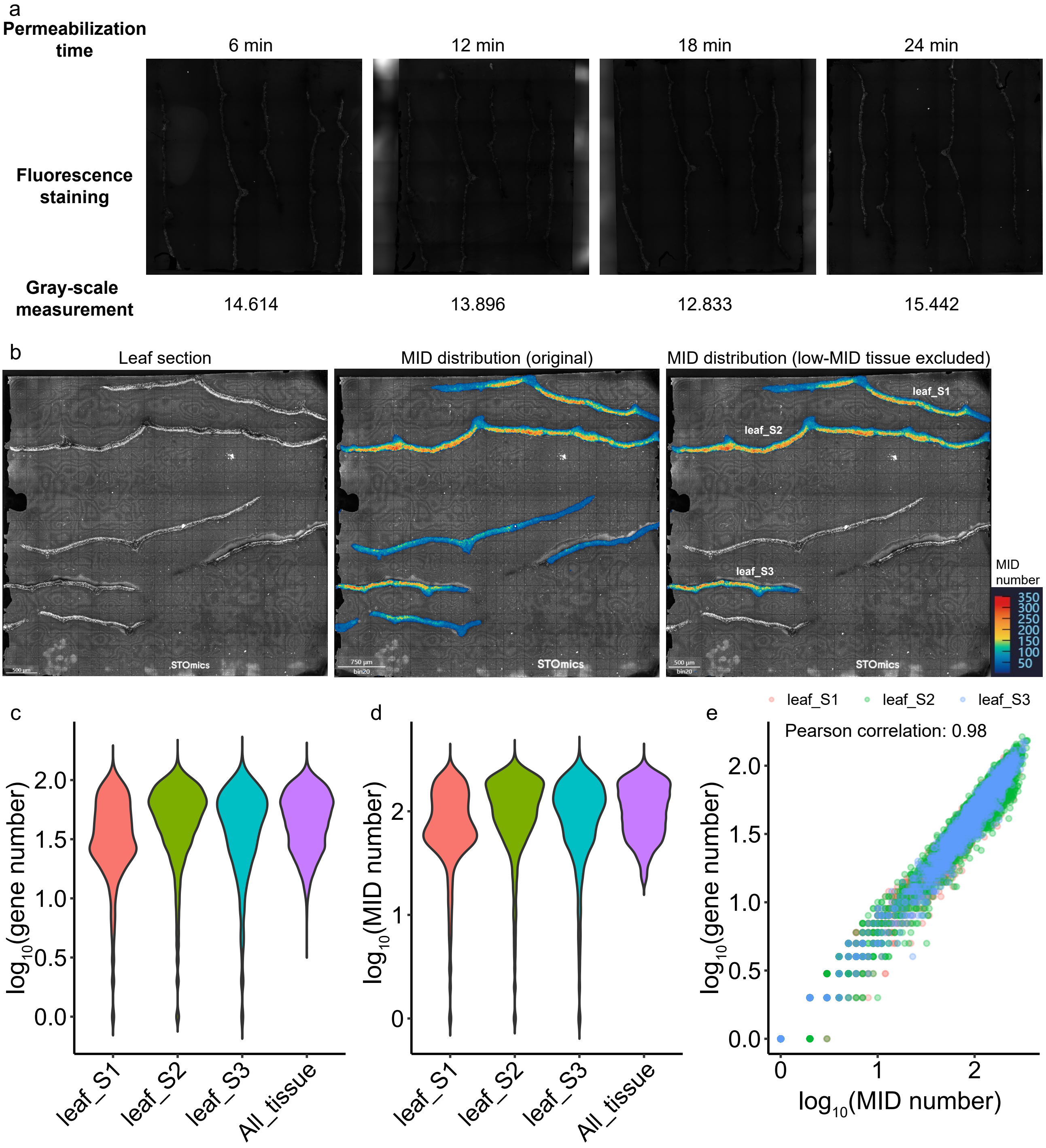

Fig. S2 Spatial RNA-seq profiling of leaf section of Cabernet Sauvignon. a. Optimization of spatial transcriptomics permeabilization conditions. Four permeabilization times were tested. 24 min is selected according to gray-scale measurement of fluorescence staining. **b.** leaves ssDNA staining picture, MID distribution in original all cauline leaf samples, MID distribution with low-MID tissue and background contamination excluded. **c. d.** Violin plots of gene number and MID number in three leaf samples and all samples together. **e.** Correlation between number of genes and MIDs in three leaf samples.

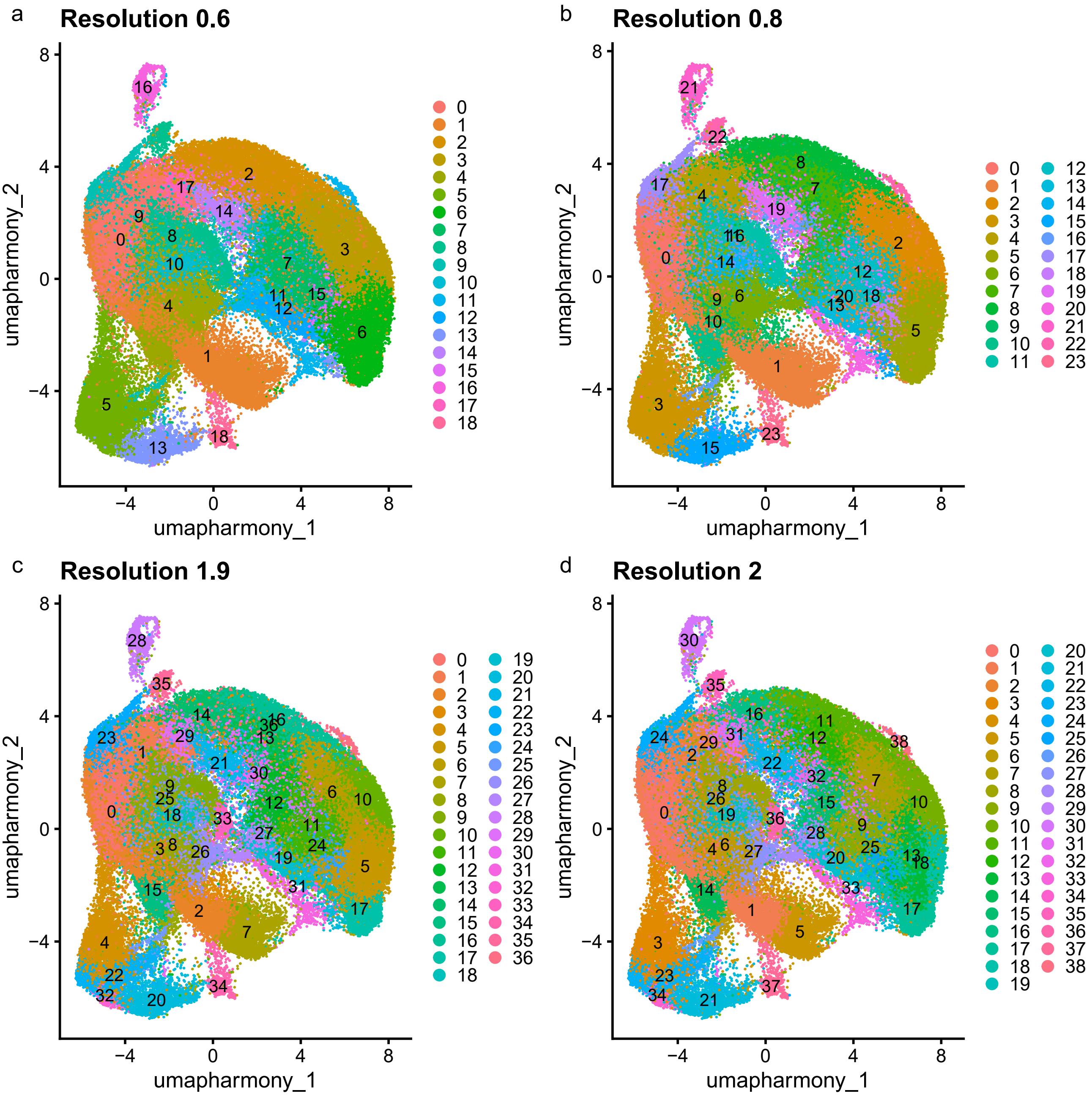

Fig. S3 Cell clustering tests for different resolution values. A series of resolution values (0.6, 0.8, 1, 1.5, 1.9, and 2, resolution 1 and 1.5 are not shown) are tested and found that a resolution of 2 yielded the most well-defined cell clusters. Notably, this resolution allowed for the clear identification of a unique cell cluster assigned to cluster 38, while other resolution values failed to identify this cluster.

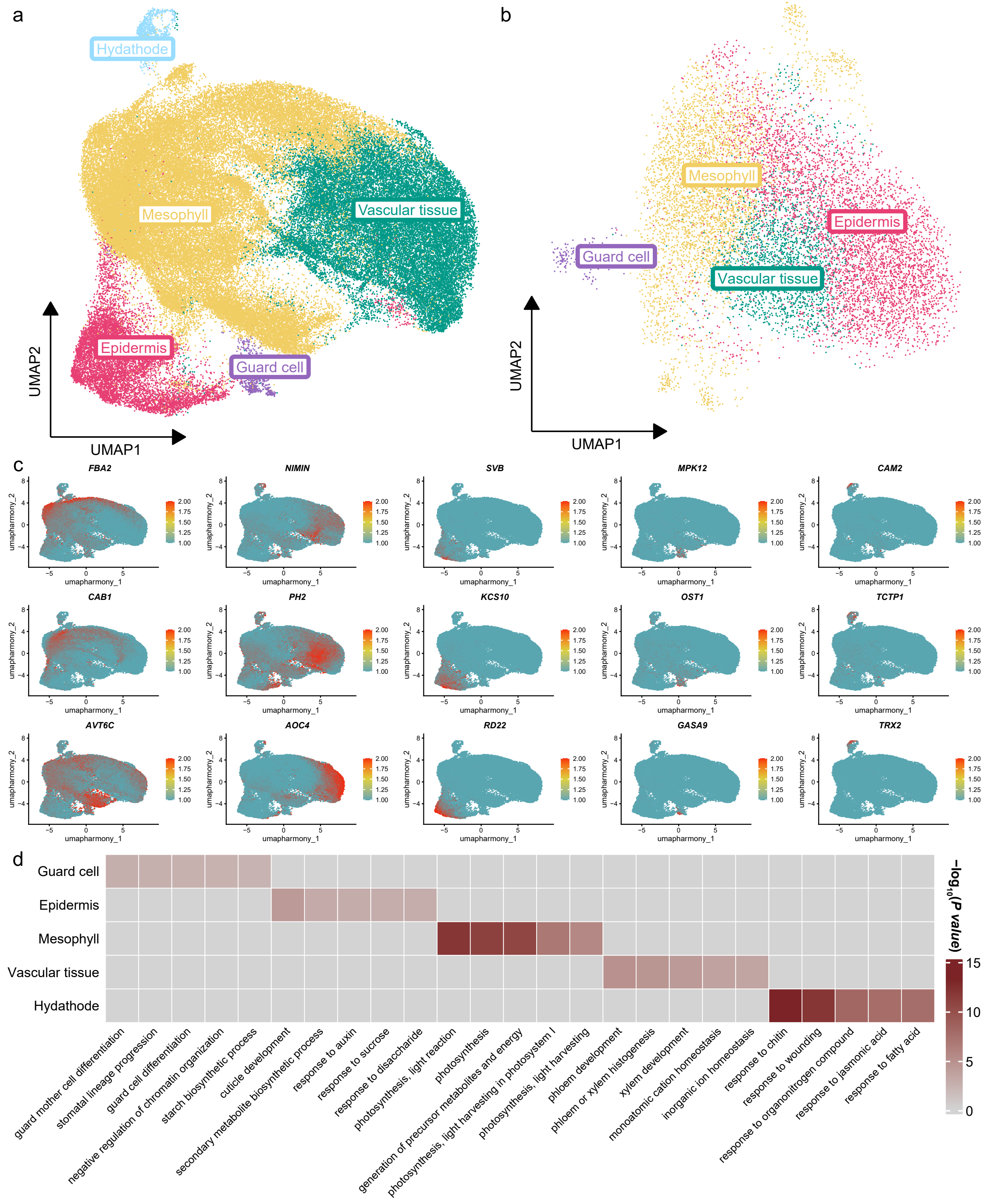

Fig. S4 Single-cell RNA-seq and spatial RNA-seq profiling with bulk tissue annotation and marker genes. a. Visualization of the scRNA-seq cell clusters using UMAP annotated based on marker gene analysis. **b.** Visualization of the spRNA-seq cell clusters annotated based on tissue location. **c.** Expression profiling of cell type marker genes including *FBA2* (mesophyll), *CAB1* (mesophyll), *AVT6C* (mesophyll), *NIMIN* (vascular tissue), *PH2* (vascular tissue), *AOC4* (vascular tissue), *SVB* (epidermis), *KCS10* (epidermis), *RD22* (epidermis), *MPK12* (guard cell), *OST1* (guard cell), *GASA9* (guard cell), *CAM2* (hydathode), *TCTP1* (hydathode), *TRX2* (hydathode). **d.** Enriched Biological Process GO terms for five different cell types.

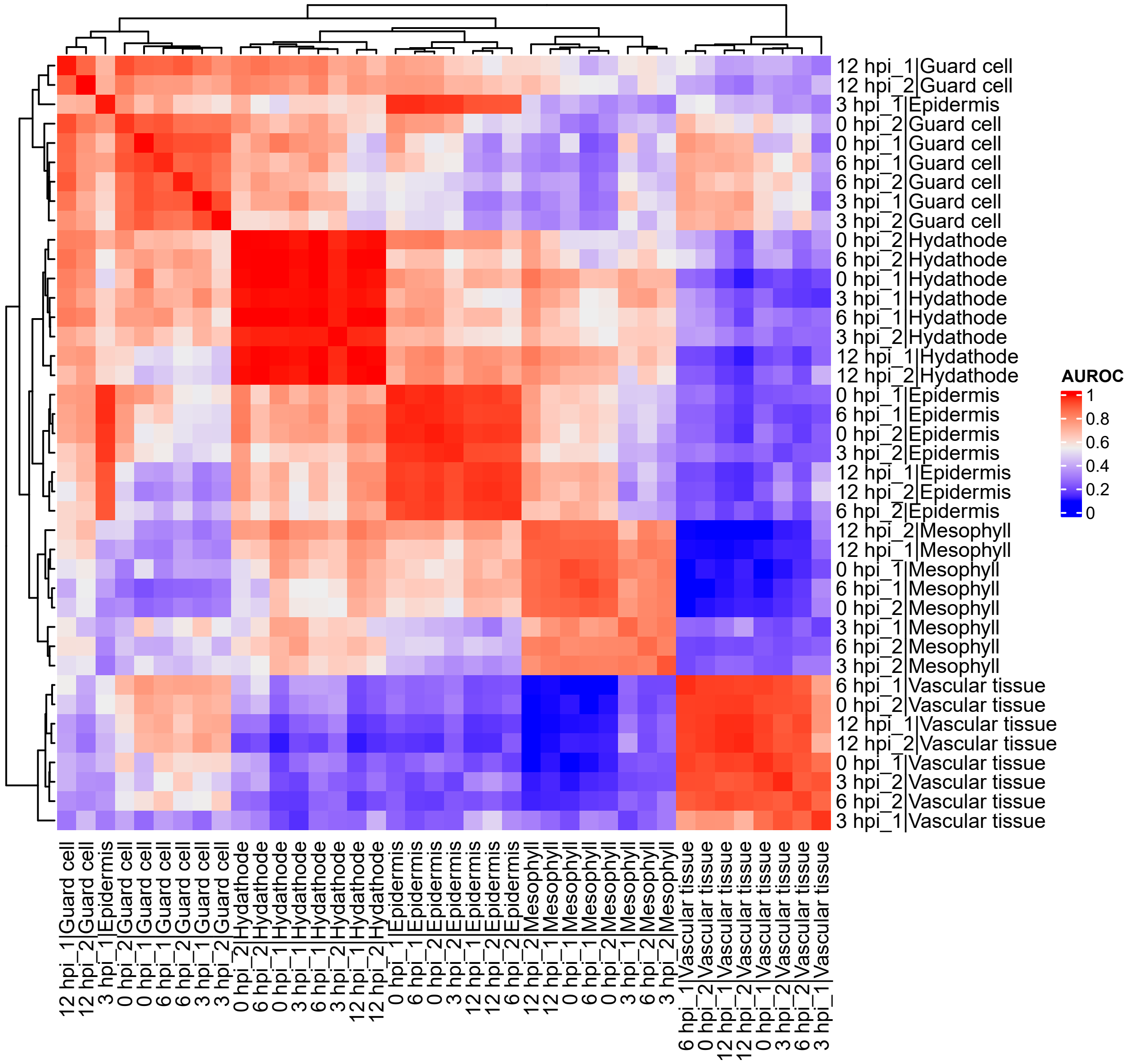

Fig. S5 Cross-library assessment of cell type annotation by MetaNeighbor. Heatmap shows AUROC scores between celltypes which were calculated based on the highly variable gene set (HVG).

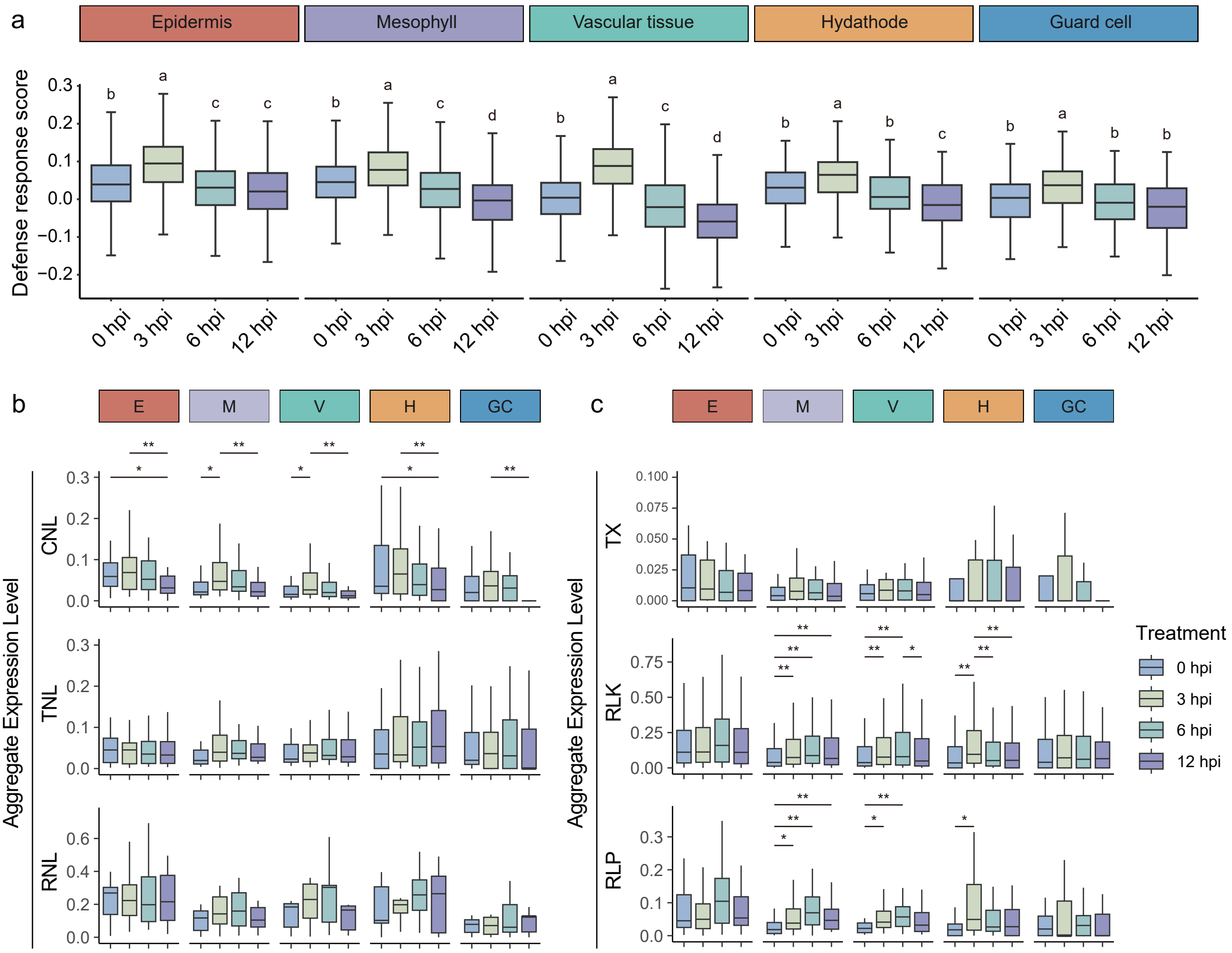

Fig. S6 Defense scores and RGAs' expression levels in different tissues. a. Quantification and multiple comparison of defense scores of four time points in five tissues. The tops and bottoms of boxes represent the 75th and 25th percentiles, respectively. Different letters above bars indicate significant differences determined using Kruskal-Wallis test with Wilcoxon rank-sum test ($p < 0.05$). **b. c.** Tissue-type-specific expression of RGAs in the scRNA-seq datasets. Gene expression levels are represented by aggregated expression level in all the cells belonging to the same cell type and in the same treatment. CNL, coiled-coil NBS-LRR; TNL, TIR-NBS-LRR; RNL, RPW8-like NBS-LRR; TX, TIR-only; RLK, receptor-like kinases; RLP, receptor-like proteins.

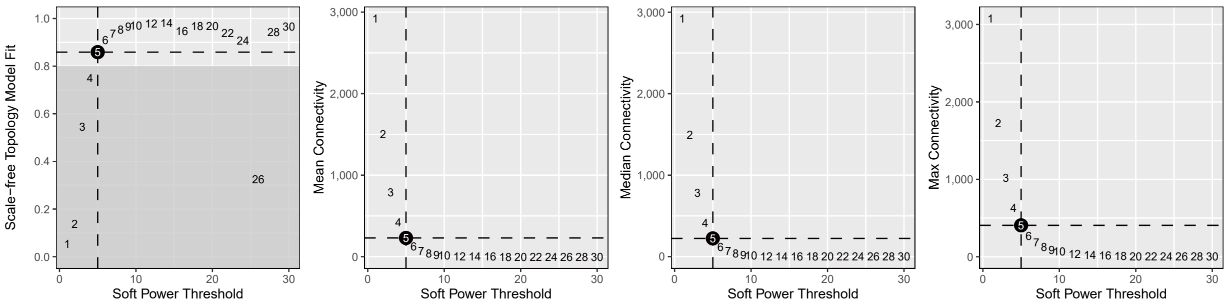

Fig. S7 Soft threshold testing for hdWGCNA. A soft threshold of 5 achieved a Scale-Free Topology Model Fit above the threshold of 0.8, while maintaining relatively high values for mean connectivity, median connectivity, and maximum connectivity.

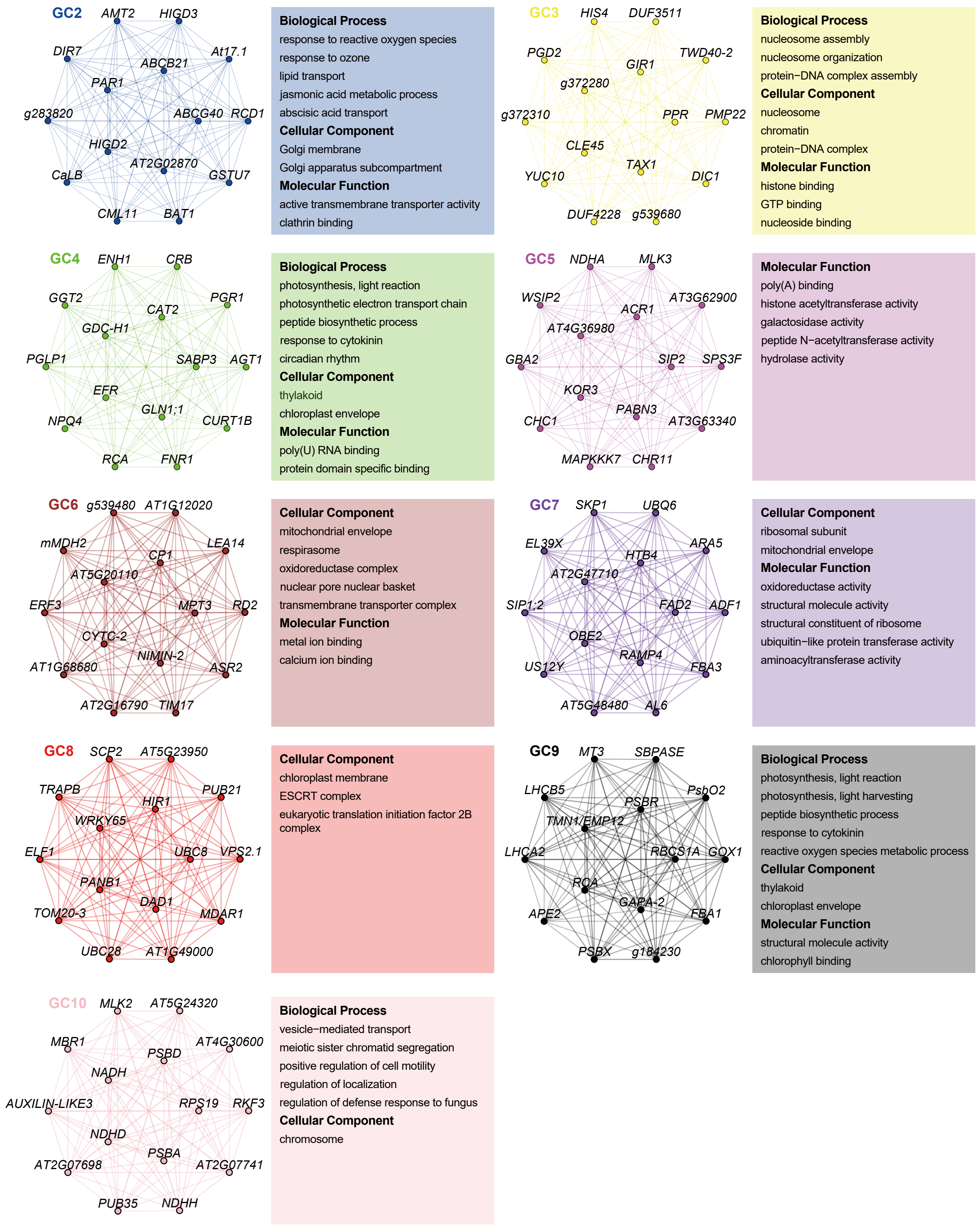

Fig. S8 GO enrichment analysis of hdWGCNA modules other than GC1. For cellular component (CC), molecular function (MF) and biological process (BP), the top five terms were shown.

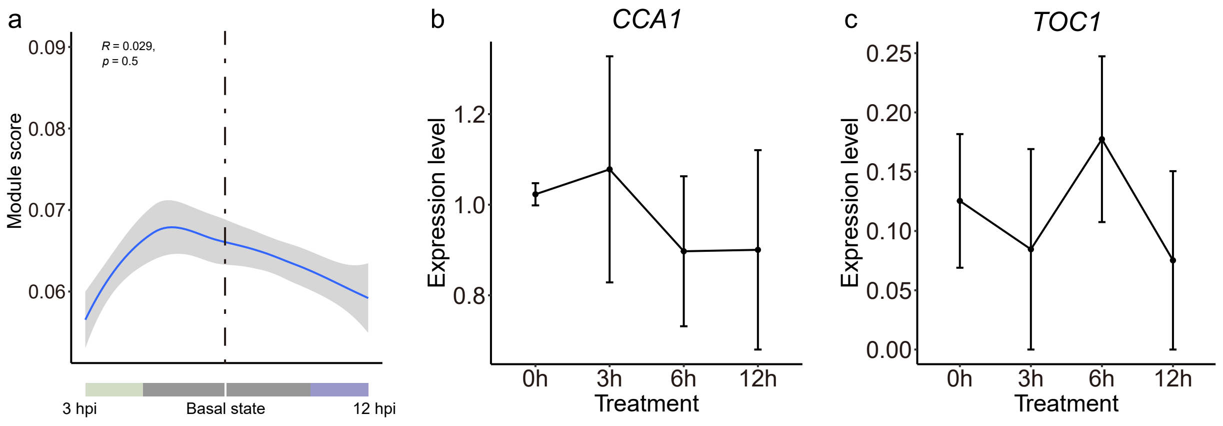

Fig. S9 Circadian rhythm pattern in guard cells during early infection stage. a. Circadian rhythms related genes in guard cells with two pseudo-time branches. The line plot shows linear regression of relative expression levels of circadian rhythms related genes. **b. c.** Expression level visualization of *CCA1* and *TOC1* in four time points extracted from pseudobulk scRNA-seq dataset.

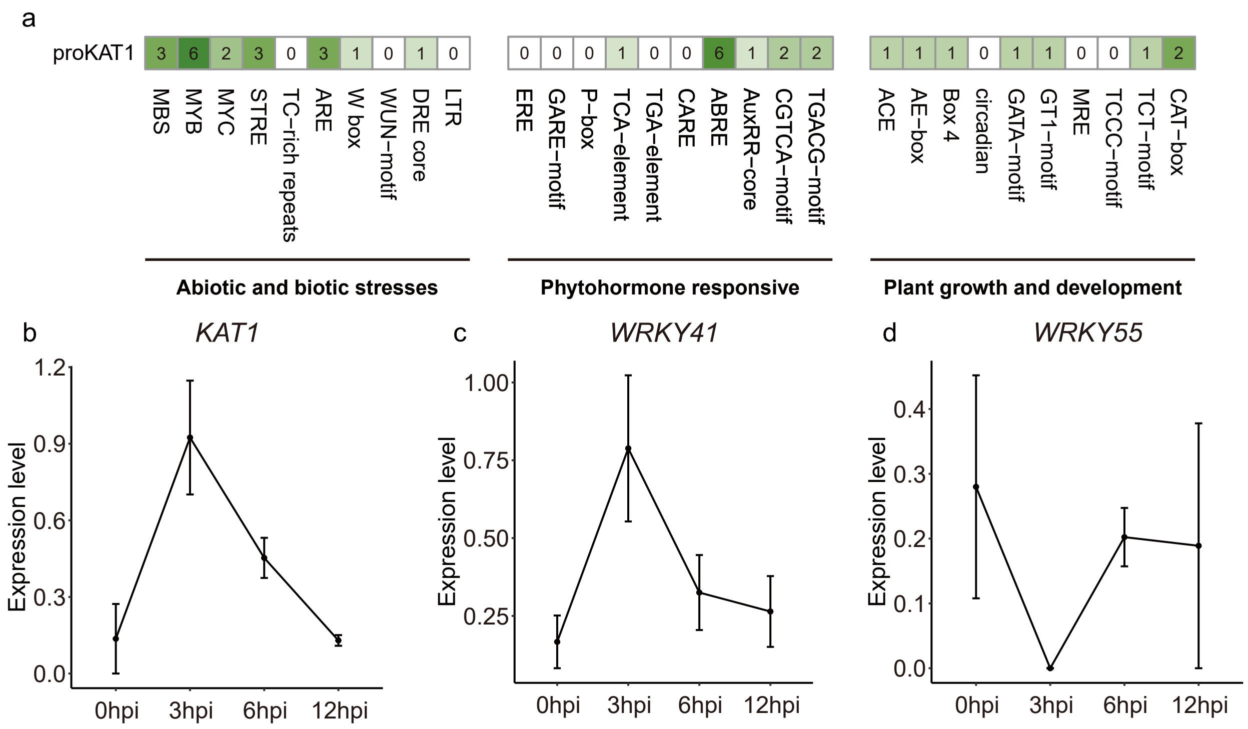

Fig. S10 Analysis of cis-acting elements on *KAT1* promoter and relative transcription factor expression level. **a.** cis-acting elements analysis on *KAT1*'s promoter, grouped into three categories based on the process they regulated. **b. c. d.** Expression level visualization of three genes in four time points extracted from pseudobulk scRNA-seq dataset. Expression level of *KAT1* had similar trends to *WRKY41*, while were opposite with *WRKY55*.
